# Effect of Primary Care Parent-Targeted Interventions on Parent-Adolescent Communication About Sexual Behavior and Alcohol Use: A Randomized Clinical Trial

**DOI:** 10.1001/jamanetworkopen.2019.9535

**Published:** 2019-08-16

**Authors:** Carol A. Ford, Jessica H. Mirman, J. Felipe García-España, Megan C. Fisher Thiel, Elizabeth Friedrich, Elyse C. Salek, James Jaccard

**Affiliations:** 1 Perelman School of Medicine, Department of Pediatrics, Division of Adolescent Medicine, University of Pennsylvania, Children’s Hospital of Philadelphia, Philadelphia; 2 Center for Parent and Teen Communication, Children’s Hospital of Philadelphia, Philadelphia, Pennsylvania; 3 Department of Psychology, University of Alabama at Birmingham, Birmingham; 4 Silver School of Social Work, New York University, New York

## Abstract

**Importance:**

Adolescent well care visits provide opportunities for clinicians to facilitate parent-adolescent communication (PAC) to reduce pregnancy, sexually transmitted infections, and alcohol-related harm among adolescents.

**Objective:**

To test the effect of brief parent-targeted interventions delivered in primary care settings on PAC about sexual and alcohol use behaviors.

**Design, Setting, and Participants:**

Randomized clinical trial conducted at a primary care pediatric practice from January 4, 2016, to April 10, 2017. Adolescents who were scheduled for a well care visit were recruited, along with their parent or guardian. Data analyses continued through April 30, 2018.

**Interventions:**

During well care visits, parents in sexual health intervention and alcohol prevention intervention groups received coaching to discuss written intervention materials encouraging PAC about sex or alcohol, respectively, with their adolescent within 2 weeks, followed by a brief clinician endorsement. After 2 weeks, parents received a follow-up telephone call. Control group parents received usual care.

**Main Outcomes and Measures:**

Participants were surveyed 4 months after the well care visit. Parent-reported and adolescent-reported quality of PAC was measured using the 20-item Parent-Adolescent Communication Scale, in which a higher score indicates better PAC; and frequency of PAC about sex or alcohol was measured using a 4-point Likert-type scale with 1 indicating not at all or never, and 4 indicating a lot or often.

**Results:**

Of 196 parent-adolescent dyads assessed for eligibility, 118 (60.2%) were eligible to participate. These 118 dyads were randomized to 1 of 3 groups: (1) sexual health intervention (n = 38 [32.2%]); (2) alcohol prevention intervention (n = 40 [33.9%]); and control (n = 40 [33.9%]); 104 parents (88.1%) and 99 adolescents (83.9%) completed the study. Parents included 112 women (94.9%) and had a mean (SD) age of 45.8 (6.9) years. Adolescents included 60 girls (50.9%); 67 adolescents (56.8%) were aged 14 years, and 51 adolescents (43.2%) were aged 15 years. Participant race/ethnicity reflected that of the practice (63 black adolescents [53.4%]; 46 white adolescents [38.9%]; 111 non-Hispanic adolescents [94.1%]). At baseline, 15 adolescents (12.7%) reported a history of sexual behavior and 16 adolescents (13.6%) reported a history of alcohol use. Intention-to-treat analyses found that 4 months after the intervention, adolescents in the sexual health intervention group reported a higher mean frequency score for PAC about sex compared with those in the control group (2.32 [95% CI, 1.97-2.66] vs 1.79 [95% CI, 1.50-2.08]; *P* = .02); adolescents in the alcohol prevention intervention group reported a higher mean frequency score for PAC about alcohol compared with those in the control group (2.93 [95% CI, 2.60-3.25] vs 2.40 [95% CI, 2.08-2.72]; *P* = .03). Parent-reported frequency scores for PAC about sex or alcohol did not differ by group.

**Conclusions and Relevance:**

Brief parent-targeted interventions in primary care settings increased adolescent-reported frequency of PAC about sexual health and alcohol use and may be an important strategy for parents to influence adolescent behaviors and health outcomes.

**Trial Registration:**

ClinicalTrials.gov identifier: NCT02554682

## Key Points

**Question:**

Can parent-targeted interventions delivered within primary care settings affect parent-adolescent communication about sexual health and alcohol use?

**Findings:**

This randomized clinical trial included 118 parent-adolescent dyads and found that brief parent-targeted interventions in primary care pediatric settings were acceptable, were feasible, and significantly increased adolescent-reported frequency of parent-adolescent communication about sexual health and alcohol use.

**Meaning:**

Delivering parent-targeted interventions in primary care settings may be an important strategy for influencing parent-adolescent communication and adolescent behaviors and improving health outcomes.

## Teaser

This randomized clinical trial evaluates the efficacy, feasibility, and acceptability of a parent-targeted intervention for parent-adolescent communication about sexual health and alcohol use.

## Introduction

Engaging in sexual behaviors and drinking alcohol during the second decade of life is common.^1,2,3,4^ Community-, school-, and home-based interventions involving direct contact between staff and parents or caregivers can favorably affect parent-adolescent communication (PAC) and a wide range of adolescent risk-associated behaviors.^4,5^ Parents can influence adolescents’ risk of unwanted pregnancy, sexually transmitted infection, and alcohol-related injury that cause substantial morbidity and mortality among adolescents and young adults.^5,6,7,8^ Enthusiasm about these interventions must be balanced with acknowledgment that such programs can be difficult to scale and sustain.

Delivering effective PAC interventions in conjunction with annual adolescent well care visits in primary care clinics could provide a recurring mechanism to systematically reach sizable portions of adolescents and their parents or guardians.^9^ This would also align with goals of patient-centered and family-centered care because adolescents and parents or guardians are interested in receiving information from primary care clinicians to facilitate increased PAC about a variety of issues, including sexual health and alcohol use.^10,11^ To our knowledge, few primary care clinic–based PAC interventions exist.^12,13^ We tested the efficacy, feasibility, and acceptability of delivering parent-targeted interventions in a primary care pediatric practice for PAC about sexual health or alcohol use. Our study was not sufficiently large or long enough to test influences of PAC on adolescent behaviors, but we adapted and tested interventions shown to improve both PAC and behavior (ie, initiation of first sexual intercourse, condom use,^14^ and alcohol-associated injury^15^) in other settings.

## Methods

### Overview

This randomized clinical trial included adolescents aged 14 to 15 years and their parent or caregiver (hereafter called *parent*) and focused on improving PAC about sexual health and alcohol use. The trial was conducted from January 4, 2016, to April 10, 2017. Data analyses continued until April 30, 2018. Another study arm involved older adolescents and focused on PAC about safe driving; that study is reported elsewhere.^16^ The study protocol (Supplement 1) was approved by the Children’s Hospital of Philadelphia Institutional Review Board. Parents provided verbal informed consent, and adolescents provided verbal assent. This study is reported following the Consolidated Standards of Reporting Trials (CONSORT) reporting guideline.

### Recruitment

Parents of all patients aged 14 to 15 years with a scheduled annual well care visit between January and September 2016 at the selected primary care pediatric practice were identified. The practice is community based and does not include trainees, and clinician salaries are linked to patient volume. Parents were mailed an introductory letter and invited to contact the study team; telephone calls were placed to all parents who did not contact the study team. Families interested in participating were screened for eligibility. The Figure presents a diagram of participant recruitment. To be eligible, adults had to be the parent or legal guardian of the adolescent scheduled for the well care visit, planning to attend the appointment, and fluent in written and spoken English. For adolescents to be eligible, they had to be aged 14 to 15 years at the well care visit, an established practice patient, fluent in written and spoken English, able to complete study procedures, and not pregnant.

**Figure.**
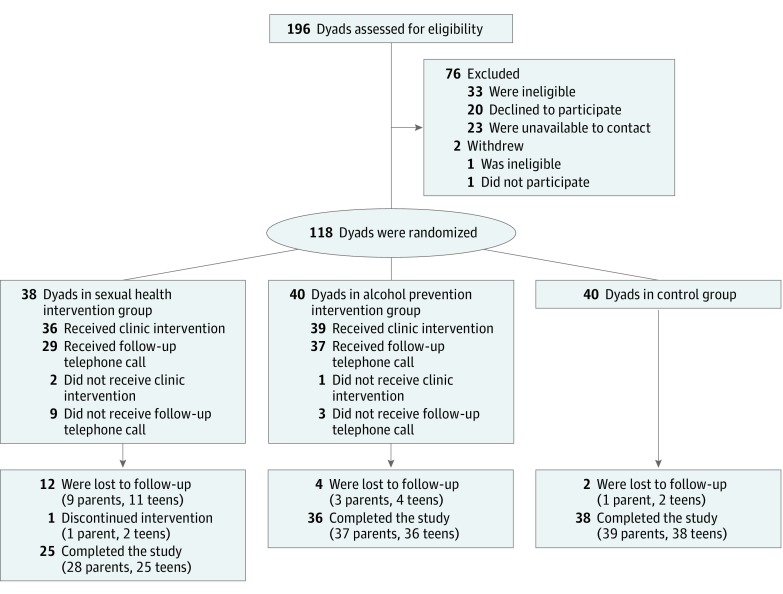
CONSORT Flow Diagram of Study Recruitment

### Procedures

Eligible parent-adolescent dyads were enrolled after providing informed parental consent and adolescent assent, and they each privately completed a telephone survey before the well care visit. The dyads were randomized into 1 of 3 groups: sexual health intervention group, alcohol prevention intervention group, or control group. Randomization was performed using a computer-generated random listing of the arms using a prespecified seed. Intervention group parents were instructed to arrive at the clinic 15 minutes early, taken to a quiet area in the waiting room away from their adolescent, and given intervention materials based on group assignment (ie, sexual health or alcohol prevention). Parents later joined their adolescent in the examination room. Parents in the control group received usual care.

Two weeks after the well care visit, intervention group parents were contacted via telephone call. Research assistants (RAs) covered material that was not completed during the in-person health coaching visit, if applicable, and administered a short survey. If parents had not had a conversation with their adolescent about the intervention topic in the preceding 2 weeks, barriers were identified and RAs discussed how the materials might be used to overcome them.

Four months after the well care visit, parents and adolescents in all groups were contacted to complete a final survey. Parents and adolescents who completed the baseline survey each received $10. Parents who completed the 2-week telephone survey received $5. Parents and adolescents who completed the final follow-up survey each received $20.

### Interventions

Intervention materials were adapted from parent-targeted interventions shown to influence PAC and adolescent sexual behavior or alcohol use. Interventions selected were informed by extensive theoretically grounded research identifying parental attitudes and beliefs affecting communication with adolescents about sexual health or alcohol use and adolescent attitudes and beliefs affecting sexual behaviors or alcohol use.^14,15,17,18^ Further, selected interventions could be pragmatically adapted for use in busy primary care settings. The intervention process included a health coach discussing written materials with parents in the clinic lobby during a well care visit, conveying key messages, and encouraging PAC within 2 weeks (Box). This was followed by a brief direct verbal and written endorsement of the intervention from the adolescent’s clinician and a 2-week follow-up telephone call from the health coach to the parent. Health coaches were college graduates without specific health care training.

Box. **Description of Parent-Targeted Sexual Health and Alcohol Prevention Interventions Delivered in Primary Care Pediatric Clinic Setting in Context of Adolescent Well Care Visits**

**Sexual Health Intervention**

**Clinic Intervention**

Health coach talks with parent in lobby to discuss:
  Sexual health brochure and handbook^a^ (provided in written form, also available in electronic form)
  General communication handbook (provided in written form, also available in electronic form)
  Key Messages:
    PAC about sexual health makes a difference
    PAC about sexual health can be hard
    Use materials to help you talk with your adolescent within 2 weeks
    Anticipated barriers to PAC and how to overcome them (ie, demonstrate how materials can be used)
    Research suggests it is important to convey these main points:
      Wait until you’re older to have sex
      If you do have sex, use condoms
      If you do have sex, go to a physician or clinic to talk about sexually transmitted infections
Physician or nurse practitioner provides a direct endorsement and written prescription reinforcing the key messages in the clinic room with the parent and adolescent at the end of the visit.

**Follow-up Telephone Call**

Follow-up telephone call to the parent at 2 weeks to ask about PAC about sexual health, inquire about barriers, and review how materials can be used to overcome barriers.

**Alcohol Prevention Intervention**

**Clinic Intervention**

Health coach talks with parent in lobby to discuss:
  Alcohol prevention brochure and handbook^b^ (provided in written form, also available in electronic form)
  General communication handbook (provided in written form, also available in electronic form)
  Key Messages:
    PAC about alcohol use makes a difference
    PAC about alcohol can be hard
    Use materials to help you talk with your adolescent within 2 weeks
    Anticipated barriers to PAC and how to overcome them (ie, demonstrate how materials can be used)
    Research suggests it is important to convey these main points:
      Wait until you’re older to drink alcohol
      If you do drink alcohol, minimize risk of injury or harm
      Do not drive or get into a vehicle with someone who has been drinking
Physician or nurse practitioner provides a direct endorsement and written prescription reinforcing key messages in clinic room with the parent and adolescent at the end of the visit

**Follow-up Telephone Call**

Follow-up telephone call to the parent at 2 weeks about PAC about alcohol, inquire about barriers, and review how materials can be used to overcome barriers.

Abbreviation: PAC, parent-adolescent communication.

^a^Content developed based on previous research identifying parental attitudes and beliefs that influence PAC about sexual health and adolescent attitudes and beliefs that influence sexual behaviors.

^b^Content developed based on previous research identifying parental attitudes and beliefs that influence PAC about alcohol and adolescent attitudes and beliefs that influence alcohol use.

Written materials were adapted to parents of our targeted age group, provided local data on sexual behaviors or alcohol use, and showed visual images reflecting the racial/ethnic composition of the local community and of gender-nonconforming adolescents and parents. Materials included colorful spiral-bound handbooks with discussion guides and activity sheets on general PAC and a similarly formatted handbook and brochure focused on PAC about sexual health or alcohol use prevention; written materials were also available electronically.

### Measures

#### Sociodemographic Characteristics

Adolescents’ age was calculated based on date of birth; sex at birth, race, and ethnicity were measured by self-report. Parental age, marital status, and highest level of education were measured by self-report.

#### Adolescent Behaviors

Adolescent behaviors were measured by self-report at baseline. Sexual behavior was assessed by 4 items: “In your lifetime, have you ever engaged in vaginal sexual intercourse? …had anal sexual intercourse? …given oral sex? …received oral sex?” Alcohol use was assessed with the question, “Have you ever had a drink of alcohol, other than a few sips?” Adolescents were coded as having engaged in adolescent risk behavior if they responded yes to any of these items.

#### Communication

There were 4 measures for reported PAC on the final survey. Quality of PAC was assessed by the 20-item Parent-Adolescent Communication Scale,^19^ in which a higher score indicates better PAC. Adolescents answered similar questions with minor word changes. Frequency of PAC about sex was measured with an item asking parents and adolescents how much they had talked about sex since the adolescent’s last well care visit using a 4-point Likert-type response categories with 1 indicating not at all or never and 4 indicating a lot or often. Frequency of PAC about alcohol was measured with a similar item asking parents and adolescents how much they had talked about alcohol since the adolescent’s last well care visit. Communication about a specific safety plan was measured by asking parents and adolescents about PAC since the adolescent’s last well care visit about strategies to avoid getting in a car being driven by someone who has been drinking, with responses coded as no communication or some PAC.

#### Intervention Feasibility and Acceptability

Feasibility was assessed by measuring length of time the RA spent with the parent (RA report) and checklists to document content delivered (ie, RA report, parent report at 2-week follow-up, and clinician report). Acceptability was assessed by final surveys. Parents were asked about general helpfulness and intentions to use materials over the next 12 months (ie, use with the adolescent participant, use with other children, or give to other parents). Adolescents were asked how helpful they thought the materials were to their parent. Clinicians received an anonymous web-based survey asking open-ended questions about implementing the study in their clinic and using these interventions in real life if they were found to be effective.

### Statistical Analysis

We tested for differences in sociodemographic characteristics between participants who completed the follow-up survey and those who were lost to follow-up. We examined frequencies of parent- and adolescent-reported PAC and evaluated bivariate associations with adolescent sex, age, race/ethnicity, and risk behaviors using analysis of variance. Unadjusted and adjusted models were estimated. Covariates in the adjusted models were adolescent age, sex, race/ethnicity, and sexual behavior or alcohol use. Generalized linear models were conducted to compare differences between each intervention group vs the control group in quality of PAC and frequency of PAC about sex or alcohol. Depending on the distribution of the outcome variable being analyzed, different distributions and link functions were specified for the generalized linear models: linear to estimate outcome means, bivariate and adjusted, with 95% CIs or log-binomial to estimate unadjusted and adjusted risk ratios (RRs) with 95% CIs. We conducted similar analyses specifically for safety plan PAC content.

Analyses were performed separately for adolescents and parents. Data were analyzed using an intent-to-treat principle and multiple imputation. To account for missing outcomes, multiple imputation with 100 imputations was used, analyses were conducted on each of the 100 imputed data set, and the parameter estimates across the data sets were combined to produce a unique point estimate and SE taking into account the uncertainty of the imputation process. Statistical analysis was performed using SAS statistical software version 14.2 (SAS Institute). Our sample size of 40 participants per group had 80% power to detect a difference in 2 population means corresponding to a Cohen *d* of 0.625, using a 2-sided *P* value of less than .05 for statistical significance. Further details on the trial protocol and statistical analysis can be found in Supplement 1.

## Results

### Sample

A total of 118 parents and 118 adolescents participated. Among parents, 112 were women (94.9%), and the mean (SD) age was 45.8 (6.9) years (Table 1). Adolescent participants were evenly split by sex (60 [50.9%] girls), and almost evenly split by age (67 adolescents [56.8%] aged 14 years; 51 adolescents [43.2%] aged 15 years). Race and ethnicity reflected practice demographics (63 black adolescents [53.4%], 46 white adolescents [38.9%]; 111 non-Hispanic adolescents [94.1%]). At baseline, 15 adolescents (12.7%) reported a history of sexual behavior and 16 adolescents (13.6%) reported a history of drinking more than a few sips of alcohol.

**Table 1.** Demographic Characteristics and Baseline Comparison by Experimental Group

Participant Characteristic	No. (%)			
	Total (N = 118)	Sexual Health Intervention (n = 38)	Alcohol Prevention Intervention (n = 40)	Control (n = 40)
**Parent**				
Women	112 (94.9)	35 (92.1)	39 (97.5)	38 (95.0)
Family structure				
Married or living with partner	91 (77.1)	30 (79.0)	29 (72.5)	32 (80.0)
Other	27 (22.9)	8 (21.1)	11 (27.5)	8 (20.0)
Highest level of education				
High school, GED, or some college	21 (17.8)	8 (21.1)	9 (22.5)	4 (10.0)
Associate or 4-y degree	36 (30.5)	10 (26.3)	12 (30.0)	14 (35.0)
Master’s or doctoral degree	61 (51.7)	20 (52.6)	19 (47.5)	22 (55.0)
**Adolescent**				
Girls	60 (50.9)	18 (47.4)	21 (52.5)	21 (52.5)
Age, y				
14	67 (56.8)	25 (65.8)	23 (57.5)	19 (47.5)
15	51 (43.2)	13 (34.2)	17 (42.5)	21 (52.5)
Race				
Black	63 (53.4)	19 (50.0)	24 (60.0)	20 (50.0)
White	46 (38.9)	16 (42.1)	13 (32.5)	17 (42.5)
Other	9 (7.6)	3 (7.9)	3 (7.5)	3 (7.5)
Ethnicity				
Hispanic	6 (5.1)	2 (5.3)	1 (2.5)	3 (7.5)
Non-Hispanic	111 (94.1)	36 (94.7)	38 (95.0)	37 (92.5)
Unsure	1 (0.9)	0	1 (2.5)	0
History of sexual behavior^a^				
Yes	15 (12.7)	4 (10.5)	4 (12.5)	6 (15.0)
No	103 (87.3)	34 (89.5)	35 (87.5)	34 (85.0)
History of alcohol use^b^				
Yes	16 (13.6)	4 (10.5)	4 (10.0)	8 (20.0)
No	102 (86.4)	34 (89.5)	36 (90.0)	32 (80.0)

Abbreviation: GED, general equivalency diploma.

^a^ Assessed by reporting to have engaged in vaginal sexual intercourse, engaged in anal sexual intercourse, given oral sex, or received oral sex in their lifetime.

^b^ Assessed with the question “Have you ever had a drink of alcohol, other than a few sips?”

The study was completed by 104 parents (88.1%) and 99 adolescents (83.9%). Fewer parents and adolescents in the sexual health intervention group were available for the 4-month interview (25 of 38 dyads [66%]) than the alcohol prevention intervention group (36 of 40 dyads [90%]) (*P* = .01) or the control group (38 of 40 dyads [95%]) (*P* = .01) (overall χ^2^ = 13.97; *P* = .001). Sociodemographic and behavior characteristics were similar between dyads who completed and those who did not complete the study.

### Bivariate Associations Between Demographic Characteristics and Communication

Responses to quality of PAC were summed into an index ranging from 41 to 96 for parents (α = .84) and 43 to 96 for adolescents (α = .87). Adolescent-reported quality of PAC varied by adolescent age; younger adolescents reported a significantly higher mean (SD) score for quality of PAC than older adolescents (72.3 [9.7] vs 66.9 [9.3]; *P* = .009). Parents of younger adolescents reported a higher mean (SD) score for frequency of PAC about sex compared with parents of older adolescents (2.7 [0.9] vs 2.2 [0.9]; *P* = .008). Parents of black adolescents reported a higher mean (SD) frequency score for PAC about sex compared with parents of white adolescents (2.7 [1.0] vs 2.1 [0.7]; *P* = .001). Black adolescents also reported a higher mean (SD) score for frequency of PAC about sex compared with white adolescents (2.2 [1.0] vs 1.8 [0.7]; *P* = .05). We found no significant bivariate associations of adolescent sex with parent-reported or adolescent-reported quality of PAC, frequency of PAC about sex, or frequency of PAC about alcohol. We found no significant bivariate associations of adolescent risk behaviors with parent-reported or adolescent-reported quality of PAC, frequency of PAC about sex, or frequency of PAC about alcohol.

### Intervention Influence on Reported Qualtiy of PAC and Topic-Specific PAC

Neither intervention influenced parent-reported quality of PAC, frequency of PAC about sex, or frequency of PAC about alcohol at the 4-month follow-up survey (Table 2). Adolescents with parents in the sexual health intervention group reported a higher mean score for frequency of PAC about sex compared with the control group (unadjusted: 2.32 [95% CI, 1.97-2.66] vs 1.79 [95% CI, 1.50-2.08]; *P* = .02; adjusted: 2.22 [95% CI, 1.84-2.60] vs 1.75 [95% CI, 1.45-2.05]; *P* = .05). Adolescents with parents in the sexual health intervention group did not report higher scores for quality of PAC or frequency of PAC about alcohol. Adolescents with parents in the alcohol prevention intervention group reported a higher mean score for frequency of PAC about alcohol during the 4 months after their last well care visit when compared with the control group (unadjusted: 2.93 [95% CI, 2.60-3.25] vs 2.40 [95% CI, 2.08-2.72]; *P* = .03; adjusted: 2.94 [95% CI, 2.56-3.29] vs 2.42 [95% CI, 2.09-2.75]; *P* = .03); they did not report higher frequency of general PAC or PAC about sex (Table 2).

**Table 2.** Effect of Parent-Targeted Interventions on Parent-Reported and Adolescent-Reported Quality of PAC and Frequency of PAC About Sexual Health and Alcohol Use

Reported Communication	Sexual Health Intervention (n = 38)		Alcohol Prevention Intervention (n = 40)		Control (n = 40), Mean Score (95% CI)
	Mean Score (95% CI)	*P* Value^a^	Mean Score (95% CI)	*P* Value^b^	
**Parent Report**					
Quality of PAC^c^					
Unadjusted	77.48 (73.89-81.06)	.98	77.27 (74.06-80.48)	.95	77.41 (74.32-80.50)
Adjusted^d^	76.49 (72.53-80.45)	.80	76.29 (72.71-79.87)	.72	77.11 (73.93-80.30)
Frequency of sex PAC^e^					
Unadjusted	2.69 (2.37-3.01)	.19	2.28 (1.99-2.58)	.57	2.40 (2.11-2.69)
Adjusted^d^	2.73 (2.40-3.07)	.18	2.29 (1.99-2.60)	.40	2.46 (2.18-2.73)
Frequency of alcohol PAC^e^					
Unadjusted	2.50 (2.18-2.82)	.96	2.58 (2.30-2.85)	.73	2.51 (2.24-2.78)
Adjusted^d^	2.61 (2.25-2.96)	.77	2.66 (2.35-2.97)	.57	2.54 (2.26-2.82)
**Adolescent Report**					
Quality of PAC^c^					
Unadjusted	72.30 (68.51-76.09)	.13	69.54 (66.37-72.72)	.65	68.50 (65.39-71.61)
Adjusted^d^	70.90 (66.81-74.99)	.32	68.71 (65.22-72.21)	.87	68.33 (65.19-71.47)
Frequency of sex PAC^e^					
Unadjusted	2.32 (1.97-2.66)	.02	2.09 (1.80-2.39)	.16	1.79 (1.50-2.08)
Adjusted^d^	2.22 (1.84-2.60)	.05	1.98 (1.65-2.31)	.29	1.75 (1.45-2.05)
Frequency of alcohol PAC^e^					
Unadjusted	2.78 (2.39-3.18)	.15	2.93 (2.60-3.25)	.03	2.40 (2.08-2.72)
Adjusted^d^	2.79 (2.34-3.24)	.17	2.94 (2.56-3.29)	.03	2.42 (2.09-2.75)

Abbreviation: PAC, parent-adolescent communication.

^a^ *P* values are of sexual health intervention group vs control group.

^b^ *P* values are of alcohol prevention intervention group vs control group.

^c^ Quality of PAC was scored using the Parent-Adolescent Communication Scale in which a higher number indicated better PAC (range: parents, 41-96; adolescents, 43-96).

^d^ Multivariable analyses are adjusted for adolescent sex, age, race, and baseline risk characteristics. Analyses were stratified by reporter.

^e^ Assessed using a Likert scale for single items with response range from 1, indicating not at all or never, to 4, a lot or often.

### Interventions’ Influence on Safety Strategy Communication

Among 40 parents in the alcohol prevention intervention group, 25 (62.5%) reported that since their last well care visit they had communicated about a specific safety strategy plan to help adolescents avoid being in a car with a driver who had been drinking alcohol compared with 13 of 40 parents (33%) parents in the control group (RR, 1.84 [95% CI, 1.25-2.42]; *P* = .001; adjusted RR [aRR], 2.02 [95% CI, 1.39-2.65]; *P* = .001) (Table 3). Similarly, adolescents with parents in the alcohol prevention intervention group were significantly more likely to report these conversations compared with adolescents with parents in the control group (27 of 40 adolescents [68%] vs 17 of 40 adolescents [43%]; RR, 1.73 [95% CI, 1.25-2.22]; *P* = .001; aRR, 1.76 [95% CI, 1.22-2.29]; *P* < .001). Parents and adolescents in the sexual health intervention group were more likely to report PAC about a specific safety strategy plan compared with those in the control group (parents: 24 of 38 [63%] vs 13 of 40 [33%]; RR, 1.79 [95% CI, 1.25-2.32]; *P* = .001; aRR, 1.75 [95% CI, 1.22-2.29]; *P* = .001; adolescents: 27 of 38 [71%] vs 17 of 40 [43%]; RR, 1.60 [95% CI, 1.15-2.06]; *P* = .001; aRR, 1.64 [95% CI, 1.20-2.09]; *P* = .001).

**Table 3.** Effect of Parent-Targeted Interventions on Parent-Reported and Adolescent-Reported PAC About Specific Safety Strategy Plan

Communication	RR (95% CI)		
	Sexual Health Intervention (n = 38)	Alcohol Prevention Intervention (n = 40)	Control (n = 40)
**Parent Report**			
Reported PAC about avoidance of getting into car with someone who has been drinking alcohol, No. (%)	24 (63)	24 (62)	13 (33)
Unadjusted	1.79 (1.25-2.32)^b^	1.84 (1.25-2.42)^c^	1 [Reference]
Adjusted^a^	1.75 (1.22-2.29)^b^	2.02 (1.39-2.65)^c^	1 [Reference]
**Adolescent Report**			
Reported PAC about avoidance of getting into car with someone who has been drinking alcohol, No. (%)	27 (71)	27 (68)	17 (42)
Unadjusted	1.60 (1.15-2.06)^b^	1.73 (1.25-2.22)^c^	1 [Reference]
Adjusted^a^	1.64 (1.20-2.09)^b^	1.76 (1.22-2.29)^c^	1 [Reference]

Abbreviations: PAC, parent-adolescent communication; RR, risk ratio.

^a^ Multivariable analyses are adjusted for adolescent sex, age, race, and baseline risk characteristics; analyses were stratified by reporter.

^b^ Statistically significant difference between sexual health intervention group and control group (*P* = .001).

^c^ Statistically significant difference between alcohol prevention intervention group and control group (*P* = .001).

### Feasibility and Acceptability

Parents spent a median of 10 (range, 7-24) minutes in coaching sessions. Among 78 parents in the 2 intervention groups, 75 (96%) received an entire health coaching session in the clinic and 74 (95%) reported receiving an in-person clinician endorsement, which was consistent with clinician reports. We were able to contact 66 intervention parents (85%) for the 2-week follow-up call.

Sixty-two parents in the intervention groups rated the intervention materials as moderately or very helpful (80%), 66 parents (85%) reported they would probably or definitely refer to materials during the next 12 months, 65 parents (83%) reported they would probably or definitely use materials with other children in their family, and 43 parents (55%) reported they would probably or definitely give materials to other parents during the next 12 months. Of the 78 adolescents in the intervention groups, 66 (85%) were aware that their parents had been given intervention materials; of these, 53 adolescents (80%) reported their parent showed them materials and 43 adolescents (65%) reported that the materials were moderately or very helpful to their parent.

Seven of 9 participating clinicians (78%) completed the feedback survey and reported materials were high quality, important, well received, and easy to disseminate. Clinicians expressed needs for additional resources (eg, staff or nurse time) to support the health coach role if interventions were to be provided in routine clinical care.

## Discussion

These parent-targeted primary care-based interventions increased adolescent-reported frequency of PAC about sexual health and alcohol up to 4 months after a well care visit. Interventions were feasible to deliver and highly acceptable to participants. Mirman et al^16^ similarly adapted a parent-targeted teenage driver safety intervention focused on older patients in the same clinic, which also demonstrated feasibility, acceptability, and initial evidence for efficacy. All interventions were adapted from interventions that, in other settings, influence PAC and adolescent behaviors associated with unintended pregnancy, sexually transmitted infections, alcohol-related injury, or motor vehicle crashes.^14,15,20^ Together, this provides strong support for future research testing the influence of pragmatic primary care–based parent-targeted interventions on PAC and adolescent health outcomes.

Several additional findings were notable. In stratified analyses, increases in frequency of PAC were reported by adolescents but not parents. This was an unexpected finding. Parents and adolescents may recognize or label PAC differently, especially about sensitive topics. Regardless, research has shown that adolescent perceptions of parental messages affect adolescent behavior, highlighting the importance of our adolescent-reported results.^21,22,23^ We observed that the reported frequency of PAC was higher for alcohol use than for sexual behavior, and more dyads in the alcohol prevention intervention and control groups were willing to complete follow-up surveys compared with those in our sexual health intervention group. This finding highlights that there are important nuances to take into consideration for PAC interventions about different topics (particularly for sensitive topics, such as sexual health) and suggests that tailoring choice of PAC interventions based on real or perceived value from the perspective of parents, adolescents, and clinicians should be considered. For example, instead of an age-based approach as used in this study, delivering PAC interventions linked to specific health topics could be informed by assessment of individual adolescent risk or adolescent or parent interest. Finally, compared with the control group, approximately 2-fold as many parents and adolescents in the intervention groups reported increased PAC about strategies for adolescents to avoid getting into a car with someone who has been drinking alcohol, although this specific content was only included in alcohol prevention intervention materials. This finding suggests that there may be broader beneficial effects of topic-specific parent-targeted interventions on PAC, which warrants further study.

It is important to note that we found these interventions feasible within the context of a research study, which supplied health coach staffing resources to provide the interventions as well as the 2-week follow-up telephone call to parents. Clinicians reported that resources for health coach staffing would be needed to implement similar strategies in real-life conditions. Our health coaches were trained in the use of materials and protocols but were not trained in health care, suggesting that it may be possible to consider a range of staffing models. Our study was intentionally designed to leverage the influence of person-to-person contact within trusted, credible clinic settings, but strategies to minimize staff time to do this could be tested (eg, text messages to replace the 2-week follow-up telephone call). Finally, future research showing that parent-targeted interventions delivered in or through primary care settings effectively change adolescent behavior and improve adolescent health outcomes would justify reimbursement for staff time to deliver interventions.

### Limitations

This study has several limitations. It was conducted at a single site, which may limit generalizability. Parent-adolescent communication was measured by self-report and could be affected by recall or self-report biases. Clinicians were not blinded in this study, which may have influenced care to adolescents in all groups. A multisite study with additional measures of PAC and a longer follow-up period is needed to evaluate intervention impact on behaviors.

## Conclusions

Results suggest that there are innovative strategic opportunities for clinicians in primary care settings to join with parents to effectively achieve better health outcomes among sizable portions of adolescent patients.^9^ The paucity of existing primary care parent-targeted interventions needs to be addressed, with a focus on developing a portfolio of interventions that effectively address a range of adolescent health issues. Developing and evaluating such interventions, and developing feasible models for incorporating them into practice, are important avenues for future research.

## Notes

Supplement 1. **Trial Protocol**

Supplement 2. **Data Sharing Statement**
