# Supplementary material for: Effect of Primary Care Parent-Targeted Interventions on Parent-Adolescent Communication About Sexual Behavior and Alcohol Use: A Randomized Clinical Trial
Source: JAMA Netw Open. 2019 Aug 16;2(8):e199535. doi: 10.1001/jamanetworkopen.2019.9535 (PMC6704744; doi:10.1001/jamanetworkopen.2019.9535)
Supplement: Supplement 1. — Trial Protocol [file jamanetwopen-2-e199535-s001.pdf]

Title: **A pilot efficacy and implementation study of the *Patients, Parents and Professionals Partnering to Improve Adolescent Health (P⁴)* Intervention**

Short Title: **P^4**

eIRB Number: 15-011732

Protocol Date: September 16, 2016

Amendment 1 Date: 9/8/2015

Amendment 2 Date: 9/28/2015

Amendment 3 Date: 10/14/2105

Amendment 4 Date: 11/9/2015

Amendment 5 Date: 12/22/2015

Amendment 6 Date: 1/8/2016

Amendment 7 Date: 2/1/2016

Amendment 8 Date: 4/11/2016

Amendment 9 Date: 4/29/2016

Amendment 10 Date: 5/19/2016

Amendment 11 Date: 6/3/2016

Amendment 12 Date: 9/16/2016

Carol Ford, MD

The Children's Hospital of Philadelphia
34th & Civic Center Blvd., 11NW10
Philadelphia, PA 19104
Phone 215-590-6864
email: fordc@email.chop.edu

TABLE OF CONTENTS

Table of Contents ii
Abbreviations and Definitions of Terms iv
Abstract v
Table 1: Schedule of Study Procedures vii
Figure 1: Study Diagram 8

1 BACKGROUND INFORMATION AND RATIONALE 9

1.1 INTRODUCTION 9
1.2 RELEVANT LITERATURE AND DATA 9
1.3 TEEN DRIVING 10
1.4 SEXUAL HEALTH 12
1.5 ALCOHOL USE / DRINKING 13
1.6 IMPROVING PARENT-TEEN COMMUNICATION IN PRIMARY CARE 13
1.6.1 Pilot Data - Acceptability 14
1.7 COMPLIANCE STATEMENT 14

2 STUDY OBJECTIVES 15

2.1 PRIMARY OBJECTIVE 15
2.2 SECONDARY OBJECTIVES (OR AIM) 15

3 INVESTIGATIONAL PLAN 16

3.1 GENERAL SCHEMA OF STUDY DESIGN 16
3.1.1 Baseline Screening Phase (T0) 16
3.1.2 Study Intervention Phase (T1) 16
3.1.3 Study Follow-Up Phase 16
3.2 ALLOCATION TO TREATMENT GROUP 17
3.3 STUDY DURATION, ENROLLMENT AND NUMBER OF SITES 17
3.3.1 Duration of Study Participation 17
3.3.2 Total Number of Study Sites/Total Number of Subjects Projected 17
3.4 STUDY POPULATION 17
3.4.1 Inclusion Criteria 17
3.4.2 Exclusion Criteria 18

4 STUDY PROCEDURES 19

4.1 SCREENING VISIT (T=0) 19
4.2 STUDY INTERVENTION PHASE 19
4.2.1 Intervention Materials 19
4.2.2 Clinic Visit (Intervention subjects only) (T=1) 19
4.2.3 Booster Call and T2 Survey (Intervention Parents only) (2 weeks) (T=2) 20
4.2.4 Final Follow-Up Survey (approximately 4-6 months) (T=3) 21
4.3 SUBJECT COMPLETION/WITHDRAWAL 21

5 STUDY EVALUATIONS AND MEASUREMENTS 22

5.1 SCREENING AND MONITORING EVALUATIONS AND MEASUREMENTS 22
5.1.1 Confirmation of eligibility 22
5.1.2 Contact Information 23
5.1.3 Baseline Screening Intake Assessment (T0) 23
5.1.4 Health Coach Acceptability, Feasibility, and Fidelity Assessment 24
5.2 EFFICACY EVALUATIONS 24
5.2.1 2 Week Survey [2 weeks post clinic visit (-3 days/+ 2 weeks)] (T2) 24
5.2.2 Final Follow-Up Survey [4-6 months post clinic visit/health coach phone call] (T3) 25
5.3 SAFETY EVALUATION 26

6 STATISTICAL CONSIDERATIONS 27

6.1 PRIMARY ENDPOINT 27
6.2 SECONDARY ENDPOINTS 27
6.3 STATISTICAL METHODS 27
6.3.1 Baseline Data 27
6.3.2 Efficacy Analysis 27
6.3.3 Safety Analysis 28
6.4 SAMPLE SIZE AND POWER 28

7 PATIENTS, PARENTS AND PROVIDERS PARTNERING TO PROMOTE ADOLESCENT HEALTH (P^4) INTERVENTION 30

7.1 DESCRIPTION 30
7.1.1 Trained RA Health Coaches (HC) 30
7.1.2 Intervention Materials 30
7.1.3 Endorsement by Health Care Provider 30
7.2 CLINICAL ADVERSE EVENTS 31
7.3 ADVERSE EVENT REPORTING 31

8 STUDY ADMINISTRATION 32

8.1 TREATMENT ASSIGNMENT METHODS 32
8.1.1 Randomization 32
8.2 DATA COLLECTION AND MANAGEMENT 32
8.3 CONFIDENTIALITY 32
8.4 REGULATORY AND ETHICAL CONSIDERATIONS 32
8.4.1 Data and Safety Monitoring Plan 32
8.4.2 Risk Assessment 33
8.4.3 Potential Benefits of Trial Participation 33
8.4.4 Risk-Benefit Assessment 33
8.5 RECRUITMENT STRATEGY 33
8.6 INFORMED CONSENT/ASSENT AND HIPAA AUTHORIZATION 33
8.6.1 Waiver of Documentation of Consent and Partial Waiver of HIPAA 34
8.7 PAYMENT TO SUBJECTS/FAMILIES 34

9 PUBLICATION 35

10 REFERENCES 36

Appendix 38

ABBREVIATIONS AND DEFINITIONS OF TERMS

AE	Adverse event
P[4]	*Patients, Parents, and Professionals Partnering to Improve Adolescent Health*
TDP	TeenDrivingPlan
ALC	Alcohol
HCP	Health care provider (physician, MD, nurse practitioner, NP)
PeRC	Pediatric Research Consortium
HC	Health Coach
RA	Research Assistant
F/U	Follow-Up

ABSTRACT

Context: Primary care provides a means by which to connect evidence-based interventions with patients; however, many interventions have been evaluated using RCTs in non-healthcare settings. Three adolescent health domains are well-suited to explore delivery of evidence-based interventions in the context of primary care: teen driving, sexual health, and alcohol use. In each of these three areas effective parent-directed interventions have been identified that measurably impact adolescent health and safety (Turrisi, Jaccard, Taki, et al., 2001; Guillamo-Ramos, Bouris, Jaccard, et al., 2011; Mirman JH, Curry AE, Winston FK, et al., 2014).

Objectives:

The primary objective is to conduct a *pilot* study to determine the effectiveness of evidence-based interventions delivered in primary care clinic settings on adolescent health behaviors and health outcomes. Key secondary objectives include assessing intervention implementation fidelity in clinical settings and examining the effect of interventions on parent-teen communication.

Study Design:

Randomized controlled trial with 2 study arms and 5 possible randomization groups [Arm 1 (14-15 year olds at well-child visit): Sexual Health, Alcohol, Control; Arm 2 (16-17 year olds at well-child visit): Driving, Control]. See Figure 1.

Setting/Participants:

The study will take place in primary care practices in the CHOP Network. Eligible participants are CHOP primary care patients that will be 14-17 years of age at their upcoming well-child visit OR 16-17 year olds that dropped off their medical certification forms for their permit application to be signed by their primary care provider. They must have a scheduled well-child visit and a willing parent participant that is planning to attend their appointment with them OR have dropped off their medical certification form in the past 8 weeks. Adolescents that are attending a *new* patient well-child visit, hold an intermediate driver's license, that are pregnant, or that have pervasive development disorder or a developmental delay are excluded from this study. Parents and adolescents must be fluent in written and spoken English as the intervention materials and health coaching is only available in English. Additionally, health care providers and research assistants (health coaches) implementing the intervention will be participants in the research study.

Study Interventions and Measures:

The study intervention being examined is called *Patients, Parents, and Professionals Partnering to Improve Adolescent Health* (P^4) and includes written materials on adolescent health topics (parent-teen communication, sexual health, alcohol, driving – inclusive of The TeenDrivingPlan Parent Guide), activity workbooks, tip sheet brochures, in person/phone health coaching, and in person or mailed health care provider endorsement and key messaging. Measures will be collected at baseline pre-intervention (T0), in clinic or over the phone at the time of the intervention (T1), two weeks post- intervention (T2), and 4 to 6 months post-intervention (T3) (See Table 1). We will measure feasibility of clinic implementation of interventions, as well as influence of interventions on parent-teen communication, parent and teen knowledge, parent and teen cognitions, and adolescent

behavioral intentions and behavior. At the conclusion of recruitment, health care providers will be asked to complete a Health Care Provider Feedback Survey.

TABLE 1: SCHEDULE OF STUDY PROCEDURES

**Study Phase**	**Baseline Screening**	**Intervention Phone Call/ Clinic Visit**	**Intervention Booster Call and Survey**	**Final Follow-up Survey**	**Final Provider Feedback Survey**
**Visit Number**	**T=0**	**T=1**	**T=2**	**T=3**	**N/A**
**Study Days**		**0**	**2 weeks**	**4-5 months (Arm 1); 6 months (Arm 2)**	
Study Letter to Families	X				
Verbal Consent to Screen	X				
Informed Consent/Assent	X				
Demographic/Intake survey	X				
Randomization	X				
Distribute Intervention Materials	X	X		X	
Clinic Orientation Session		X			
HCP Endorsement		X			
Booster Call and Survey			X		
Final Follow-Up Survey				X	
Adverse Event Assessment		X	X	X	
Quality Improvement Survey with HCPs					X

FIGURE 1: STUDY DIAGRAM

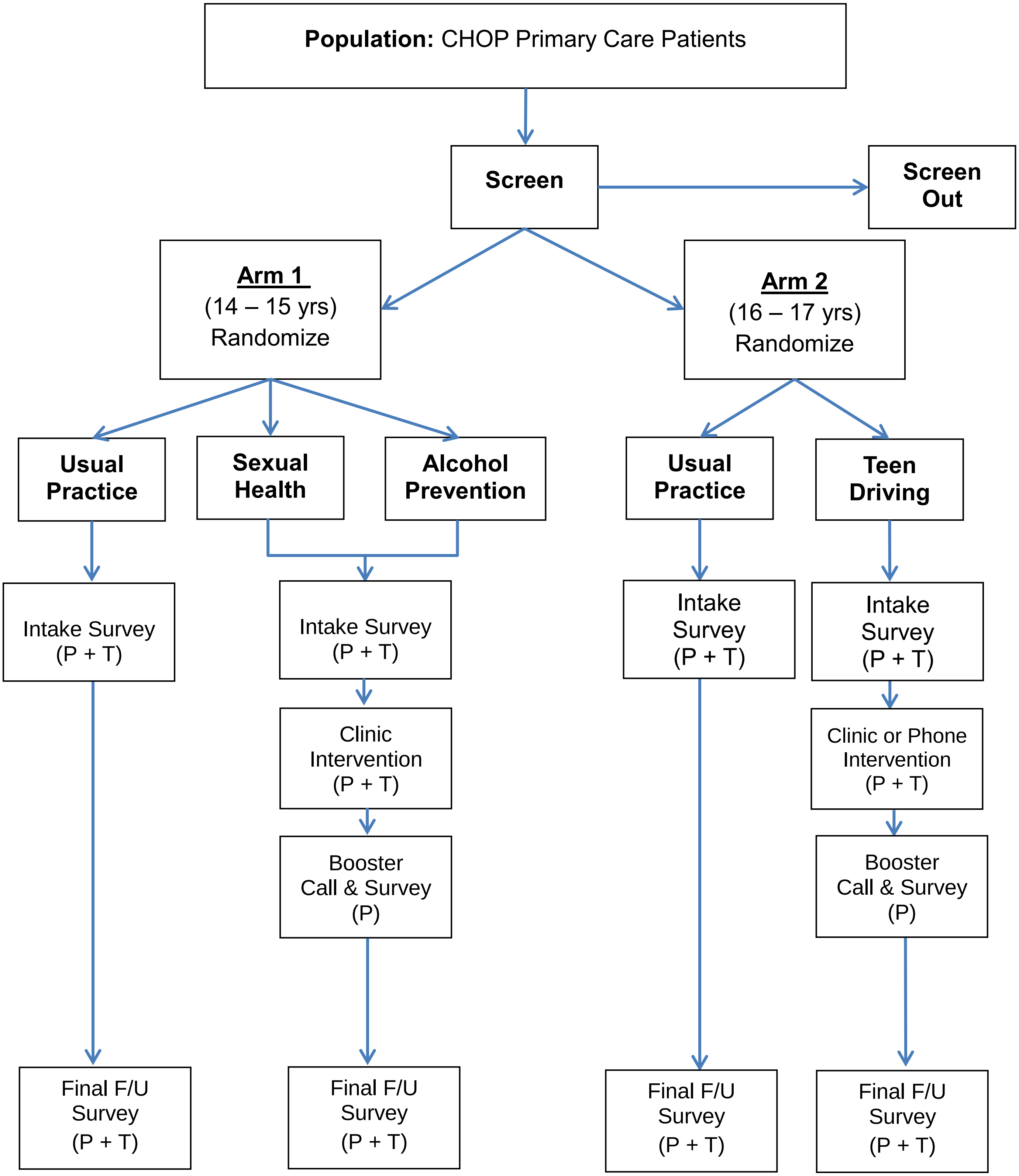

*P = Parent; T = Teen

1 BACKGROUND INFORMATION AND RATIONALE

1.1 Introduction

Prior research has found that relationships between health care professionals (HCP)'s, parents and adolescent patients can be enhanced directly and indirectly (Ford et al., 2011). Direct strategies involve strengthening relationships and/or communication between parents and HCPs in both practice and community settings. Indirect strategies involve opportunities for HCPs to influence parent–adolescent relationships and/or communication within the context of adolescent visits. For example, HCPs can discuss the importance of parental involvement and monitoring with adolescents, encourage and facilitate parent–adolescent communication, and deliver tailored parental guidance (Ford et al., 2011).

Primary care practices provide a means by which to connect evidence-based interventions with patients and parents; however, many interventions have been evaluated using RCTs in non-healthcare settings. Implementing (and/or disseminating) and studying these interventions in health care settings, such as primary care, will require additional research concerning implementation fidelity and efficacy.

1.2 Relevant Literature and Data

An evidence-informed conceptual model for intervening with HCPs and parents is presented in Figure 2. As depicted in this figure, HCPs can provide general and individualized (i.e., tailored information) to parents in order to improve adolescent health. More specifically, HCPs can endorse evidence-based interventions and provide a pathway to obtain evidence-based interventions for parents and teenagers. Coupled with the endorsement of the HCPs, parent-directed interventions to improve adolescent health might gain potency. As depicted in Figure 2, armed with their new knowledge and skills, parents can alter the content and nature of their communications with their adolescent, provide novel resources and domain-specific support, and continue to monitor with warmth, and encourage autonomy to promote overall health and safety.

Many adolescent health domains are well-suited to explore delivery of evidence-based interventions in the context of primary care. In the current study we will examine three topic areas: teen driving, sexual health, and alcohol use. In each of these three areas effective parent-directed interventions have been identified that measurably impact adolescent health and safety (Turrisi, Jaccard, Taki, et al., 2001; Guillamo-Ramos, Bouris, Jaccard, et al., 2011; Mirman JH, Curry AE, Winston FK, et al., 2014). Pathways for delivery of these interventions in health care settings that ensures implementation fidelity are lacking. Additionally, these interventions do not contain administrative content directed specifically to HCPs. Developing and evaluating this new content for implementation and efficacy will be critical for effective deployment.

Figure 2. General framework for parent-HCP partnerships to improve adolescent health

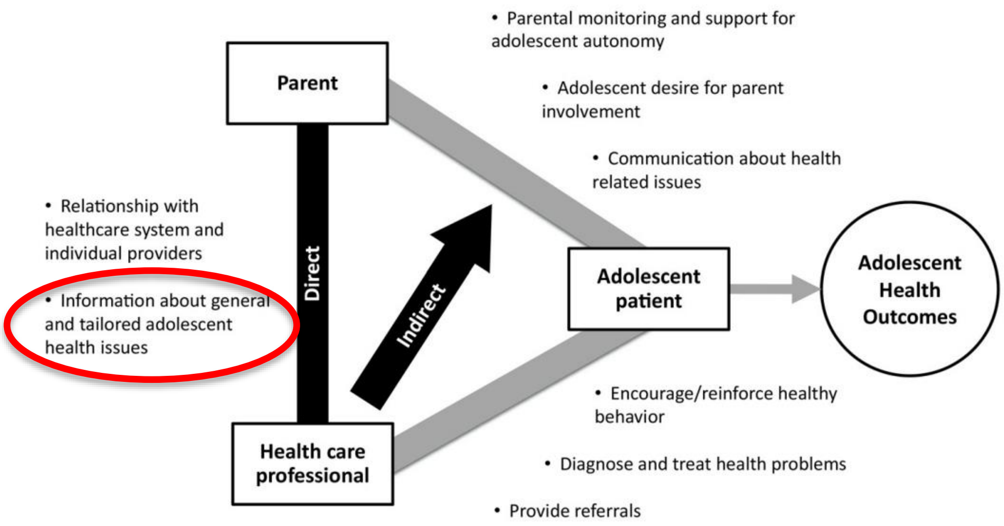

1.3 Teen Driving

Motor vehicle crashes remain the leading cause of death among teens in the United States. Teen drivers (ages 16 to 19) are in fatal crashes at four times the rate of adult drivers (ages 25 to 69). Inexperience is a fundamental factor in the high crash rate for novice teens. Studies have shown that novice drivers are at highest risk for crashing within the first six months post-licensure; *by contrast, the lowest lifetime risk of crashing occurs when the teen is driving while accompanied by an adult as a "learner driver"* (Mayhew, Simpson, & Pak, 2003; McCartt, Shabanova, & Leaf, 2003). During the learner period, teens ride with both family members and instructors. Many but not all teens learn driving skills through a minimal amount (on average, six hours) of behind-the-wheel lessons with instructors. Nearly all teens learn to drive with adult family members (usually parents). The learner period provides an opportunity to teach, practice and evaluate whether the teen has the behaviors and skills necessary to ride without adult supervisions. Unfortunately, the quantity, quality and diversity of practice driving is very limited and many teens start unsupervised, post-licensure driving without sufficient preparation.

Safe and skilled driving is a complex task that involves behaviors (e.g., skills and actions) that may vary by driving environment. Scanning, for example, is such a behavior. On neighborhood roads, the driver needs to learn how to scan for other road users (e.g., bicyclists) or unexpected hazards (e.g., a child darting out in the street). In contrast, highway driving requires the driver to learn how to scan far ahead in order to detect changes in driving conditions in sufficient time to slow or stop from a high speed. These behaviors are learned through instruction and a considerable amount of experience. Ideally, the behaviors are first taught by a professional (e.g., a driving instructor) while families provide the necessary hours of practice. Varied, high quality practice driving with an adult family member for a sufficient number of hours can provide teens with a safe way to gain needed experience that will prepare him/her to ride safely without supervision once licensed.

The TeenDrivingPlan.org (TDP) has been designed to help improve the quantity and quality of parent facilitated-practice driving through guided learning, planning and logging of practice drives. More specifically, the program encourages parents to increase the number

of hours of behind-the-wheel (BTW) practice and to increase the range of environmental conditions and driving behaviors explicitly practiced during those hours. The program provides targeted on-line education and tools for planning and logging. All choices and plans for practice driving activities are made by the families.

The program integrates practical information for parents and teens with evidence-based intervention components to promote practice driving. It includes text-based information; videos, graphical and audio materials; and interactive planning and logging tools. TDP is a tool to provide families with a structure for facilitating practice drives by grouping road types into progressively complex environments in which teens will practice specific driving behaviors and skills. It presents information organized around driving environments, which include a large empty parking lot, residential neighborhoods, commercial roads, intermediate and rural roads and highways, as well as challenging driving situations such as driving at night and in inclement weather.

Content was identified using a variety of research methods including literature review, parent and teen focus groups, a 2010 process evaluation study (IRB 10-007530) of an early, limited version of TDP, interviews with international scientific driving experts, and interviews with a panel of expert driver education instructors. Creative content was developed and refined using input from parents, teens, and driver education instructors. Usability testing informed the design and functionality of the web application. Results from a randomized controlled trial are shown in Table 2 (IRB 11-008203) (Mirman, Curry, Winston, et al., 2014; Mirman, Albert, Curry, et al., in press; Winston, Mirman, Curry et al., 2014).

Table 2. Key Results of the TDP Evaluation (Randomized Controlled Trial)
(1) **Driving Skill:** Teenagers in the TDP group were 65% less likely to fail a rigorous on-road driving assessment. The Hazard Ratio for exposure to TDP was 0.35 (95% CI, 0.12-1.03; log-rank p = 0.05). Six percent of teenagers that used TDP had their on-road driving assessment terminated due to unsafe driving performance as compared to 15 percent of those not given TDP (risk difference [TDP-control]: -9% (-21%, 2%), p = 0.06).
(2) **Parent Supervision:** TDP parents were more engaged supervisors (e.g., regularly logged drives, gave feedback) (parent *and* teen report), p = 0.03 and TDP teens reported greater instrumental and socioemotional support from parents related to practice driving (teen report), p = 0.01.
(3) **Practice Variety:** TDP dyads practiced at least 1-2 hours in a greater variety of driving environments than control dyads, p = 0.01 (parent *and* teen report). This increase in variety mediated the effect of TDP on driver performance.
(4) **TDP Use:** The estimated relative rate (RR) of TDP use (valid log-ins) was three times higher (3.08, 95% CI 1.67 to 5.69) during the first week among dyads who achieved high practice diversity compared to dyads who practiced in fewer environments. At week 3, the RR was 1.55 times higher (95% CI 0.99 to 2.43); use was similar by week 5.

Stemming from this success, a novel implementation of an adapted version of TDP in the primary care setting in conjunction with providers' completion of the state of Pennsylvania's driver's license permit form (DL-180 (3-14)) could offer several advantages. This form requires qualified health care providers to affirm that there are no medical or psychiatric conditions (e.g., uncontrolled diabetes, alcohol or drug abuse, other cognitive impairment) that preclude the teenager from safely pursuing licensure. Completion of this form could be

leveraged to support the provision of evidence-based tools and interventions for all families. However, there are no best-practice guidelines that support completion of this form, which is supposed to be signed by the applicant in the presence of the provider and no interventions directly connected to supporting this critical provider-patient-parent interaction at the outset of adolescent patients entering their highest lifetime risk for a MVC.

1.4 Sexual Health

According to the Youth Risk Behavior Surveillance (CDC, 2013) 46.8% high school students reported previously having sexual intercourse and 34.0% had intercourse in the previous 3 months (i.e., were “currently” sexually active). Nearly 41% of students who had sex in the prior 3 months did not use a condom the last time they had sex, 15.0% had at least four or more sexual partners, 19% reported that they (or their partner) used birth control pills to prevent pregnancy at the time of last sexual intercourse, while 14% reported using no form of birth control. Further, almost 23% had used drugs or alcohol before their last time of sexual intercourse. Collectively, these behaviors increase adolescents’ risk for sexually transmitted infections (STIs) and unintended pregnancy (CDC, 2009; Weinstock, Berman & Cates, 2004; Hamilton, Martin & Ventura, 2009). Delaying the time of first sexual intercourse and promoting safe sexual practices including using birth control and regular STI testing among sexually active youth is paramount for preventing STIs and unintended pregnancies.

A variety of methodologies have been employed to encourage adolescents to adopt safe and responsible sexual practices to youth of varying levels of risk. Parent-directed approaches offer several unique benefits compared to group-based or school-delivered programs including the opportunity for parents to communicate information consistent with their personal values, tailor information to be responsive to the child’s life circumstances and contexts and their child’s developmental level (e.g., cognitive, social and emotional), and bundle these communications into a broader set of parent-child interactions related to monitoring and surveillance (Jaccard, Dodge, Dittus, 2002).

A clinic-based RCT (n=264 dyads) was conducted to evaluate the efficacy of *Families Talking Together*, a parent-based intervention to prevent sexual risk behavior, among Latino and African American male and female teenagers between the ages of 11 to 14 years and their mothers, compared to a standard care control condition (Guillamo-Ramos, Bouris, Jaccard et al., 2011). Intervention components were delivered to mothers in waiting rooms during their teenagers’ clinic visits. Dyads completed a brief baseline survey in the clinic, were randomized and then completed a follow-up survey nine months later in the home. Primary outcomes included whether the adolescent had ever engaged in vaginal sexual intercourse (yes or no), and among the sexually active respondents, the frequency of sexual intercourse, and the frequency of oral sex were ascertained. The parent-directed intervention consisted of 4 key components: (1) a 30 minute face-to-face meeting with a social worker; (2) a written informational “manual” on effective communication and parenting strategies for reducing adolescent sexual risk behavior with two activity guides; (3) booster phone calls to direct parents to the intervention materials and 4) provider endorsement of the materials. Findings from the RCT found that sexual activity was attenuated in the intervention group (6%) and increased from 6% to 22% for young adults in the control condition. Incorporating this effective intervention into the workflow of a busy primary practice requires considering how to repackage successful intervention components and materials in a cost-effective and scalable way.

1.5 Alcohol Use / Drinking

According to the Youth Risk Behavior Surveillance (CDC, 2013), 66.2% of high school students nationwide had at least one drink of alcohol on at least 1 day during their life and 34.9% of high school students had drunk alcohol in the previous 30 days. 21.9% of students nationwide had ridden in a car driven by someone who had been drinking alcohol and 10.0% (of the 64.3% of youth who drove in the last 30 days) had driven when they had been drinking alcohol. Nationwide, 20.8% of students had had five or more drinks of alcohol in a row.

Data from the National Longitudinal Study of Adolescent Health indicates that problem drinking occurs in youth as early as seventh grade and that young people are more likely to exhibit binge drinking behaviors if they report poor parental communication and monitoring (Guilamo-Ramos, Jaccard, Turrisi et al., 2005). Thus, one potential strategy for dealing with alcohol problems in youth is by improving parental communication about and monitoring of alcohol related behaviors. A particularly promising parent-based intervention attempted to influence students before they start college through their parents (Turrisi, Jaccard, Taki, et al., 2001). Specifically, parents received informational materials about the prevalence and risks associated with binge drinking and how to convey information about drinking to their teens. Parents were encouraged to talk with their teens prior to leaving for college. Teens whose parents implemented the intervention materials exhibited reduced drinking tendencies and experienced fewer drinking-related consequences.

1.6 Improving Parent-Teen Communication in Primary Care

Research shows that parents play an important role in their child’s health. In particular, parent-based interventions that seek to improve parent-teen communication have been shown to be effective at reducing adolescent risk taking and improving adolescent healthy decision making. According to Jaccard, Dodge and Dittus (2002) parent-based approaches allow parents to: (1) discuss information in a way that is consistent with their values, (2) alter the presentation of information to fit social and familial contexts, and (3) increase supervision of teenagers’ activities. Additionally, previous studies have shown parent-teen communication to be an important factor in reducing adolescent risk taking behaviors. One clinic-based RCT that evaluated a parent-based intervention to reduce sexual risk behavior in early adolescence found that sexual activity remained at 6% among adolescents in the intervention condition, but increased from 6% to 22% for adolescents in the “standard of care” control condition (Guillamo-Ramos, Bouris, Jaccard et al., 2011). Similarly, a parent-based intervention designed to influence college drinking behaviors was shown to reduce drinking and tendencies toward drunkenness, increase negative perceptions toward drinking activities, reduce peer and parental approval of drinking, and decrease drinking-related consequences (Turrisi, Jaccard, Taki, et al., 2001). Additionally, parents identified parent-teen communication as an important factor in keeping teens healthy in a small, exploratory study examining the partnership between parents and health care providers. Parents were asked: *What can parents do to keep teens healthy and address health problems?* 47% of parents mentioned parent-teen communication, citing the importance of talking, listening and giving encouragement (Ford, Davenport, Meier et al., 2009). Our own pilot work confirmed that parents are interested in getting information from their child’s primary health care provider about how to communicate with their child about a range of health behaviors (Ford et al., manuscript in preparation). Primary care is an ideal location to intervene on parent-teen communication as it is protected time for discussing important teen health issues and it involves a trusted expert, who can highlight the importance of

parent-teen communication about teen health issues and endorse evidence-based interventions for parents to continue discussions at home.

1.6.1 Pilot Data - Acceptability

During qualitative pilot work (IRB # 14-010850), we investigated adolescent and parent interest in receiving information about health topics and parent-teen communication from clinicians (Ford et al., manuscript in preparation). 91 parent-adolescent dyads completed individual interviews assessing their levels of interest in receiving health and health communication information from the adolescent's doctor about a variety of topics, including routine, mental health, sexual health, substance use, and injury prevention issues. The sample consisted of mostly female parents (84%) and adolescents that were evenly divided by gender and age; 36 were 12-13, 35 were 14-15, and 20 were 16-17 years of age. Adolescent race reflected the practice population (60% black; 35% white). Results indicated that the majority of parents and adolescents reported moderate or high levels of interest in receiving information about all health issues, inclusive of information to increase parent-teen communication. From this preliminary work, it is clear that both parents and adolescents want health care professionals to help them learn, and talk about, a wide range of adolescent health topics. Thus, primary care interventions that effectively improve parent-teen health communication and specific adolescent health outcomes would be of value to parent and teens.

Additional pilot work was conducted to determine acceptability of the intervention materials prior to trialing them (IRB #15-011780). We recruited 68 parents of adolescent CHOP PBRN patients to review and provide feedback on intervention materials (parent-teen communication, targeted health topics) and study procedures. The study sample primarily consisted of caregivers who were mothers (90%), had a 4-year degree or more (64%), were married (65%) and had an average of 2 children. About half (54%) of the caregivers were African American followed by white (43%); few caregivers were Hispanic (2%). 92% regularly wear a seatbelt when they drive a vehicle and 78% regularly use the internet or check email several times a day from home. In general, participants liked the materials and cited several benefits including: "opening the door to having a dialogue with your teen", "getting on the same page as their teen's doctor", and "sending a consistent message to the teen." Parents reported a strong likelihood that they would use the materials and expressed a strong desire to participate in a related research study (unpublished results; contact Jessica Mirman for more details).

1.7 Compliance Statement

This study will be conducted in full accordance with all applicable Children's Hospital of Philadelphia Research Policies and Procedures. All episodes of noncompliance will be documented.

The investigators will perform the study in accordance with this protocol, will obtain consent and assent, and will report unanticipated problems involving risks to subjects or others in accordance with The Children's Hospital of Philadelphia IRB Policies and Procedures and all federal requirements. Collection, recording, and reporting of data will be accurate and will ensure the privacy, health, and welfare of research subjects during and after the study.

2 STUDY OBJECTIVES

The purpose of the pilot study is to determine implementation fidelity and efficacy of parent-based interventions conducted in a primary care practice.

2.1 Primary Objective

The primary objective of the study is to determine if the intervention(s) affect teen health behaviors and cognitions.

2.2 Secondary Objectives (or Aim)

The secondary objectives are to: (1) assess the impact of the interventions on the quality of parent-teen communication and their knowledge about important teen health behaviors; (2) assess the fidelity of implementation of the interventions in clinic settings; and (3) to conduct exploratory analyses to refine the theoretical model on which the interventions are based.

3 INVESTIGATIONAL PLAN

3.1 General Schema of Study Design

This is a pilot randomized controlled trial to assess efficacy, estimate clinic-level effect sizes on key outcomes, and assess the implementation of the P^4 intervention. See Figure 1 and Table 1 for overviews.

3.1.1 Baseline Screening Phase (T0)

Potential subjects will be identified using EPIC as all will come from participating PeRC primary care practices. Additionally, 16-17 year old patients will be identified by the participating primary care practice when patients drop off their medical certification form for the permit application to be signed by their CHOP primary care provider. A letter will be given or sent in the mail to all eligible families to describe the study and give them the opportunity to call or email the study team for more information about participating. They will be verbally screened and consented using the protocol inclusion and exclusion criteria into two strata based on age at upcoming well-child visit (Arm 1: 14 to 15 years and Arm 2: 16 to 17 years) OR current age (16-17) if teen recently had medical certification form signed. We are requesting a waiver of assent for screening procedures, however parental consent and child assent will be obtained over the phone for the main study procedures. We are also requesting a waiver of written documentation (see Section 8.6.1). After randomization into a study group, a demographic and baseline intake survey will be completed with the parent and teen over the phone and the educational materials will be sent electronically (Arm 2 only), mailed (Arm 2 only), and/or given in person to the intervention group parents after enrollment. For a full list of assessment instruments see section 5.0.

3.1.2 Study Intervention Phase (T1)

The study intervention involves educational materials for parents to read, an in person or phone orientation to the intervention materials delivered by a trained Research Assistant (RA), as well as a brief endorsement of the materials by the health care provider (HCP) during the teen's well-child care appointment or via a provider "prescription" mailed to the parent, and a booster health coach call delivered by a trained RA. The RA will complete a fidelity assessment after the health coaching clinic visit or phone call, and booster call to document the components of the intervention that were delivered.

3.1.3 Study Follow-Up Phase

3.1.3.1 T2

A survey will be administered to parents in the intervention groups via phone or a secure web survey platform within 2 weeks (- 3 days/ + 2 weeks) of the health coaching clinic visit or health coaching phone call. Surveys can be conducted at the end of the booster call or will be scheduled for another date depending on parent availability. For a full list of assessment instruments see section 5.0.

3.1.3.2 T3

Electronic and/or phone follow-up surveys will be collected with all parents and teens in Arm 1 approximately 4-5 months after the well-child appointment and with all parents and teens in Arm 2 approximately 6 months after the well-child appointment or health coach phone call. For a full list of assessment instruments see section 5.0.

3.2 Allocation to Treatment Group

Randomization will be performed using a table of random numbers generated at the outset of the study. Neither the RA enrolling the dyad or the parent/teen dyad who agrees to participate will be blind to the dyads' allocations. During screening, participants will be allocated into two strata based on age at upcoming well-child visit (Arm 1: 14 to 15 years and Arm 2: 16 to 17 years) (See Figure 1). 16-17 years old who drop of their medical certification forms for the permit application will automatically be allocated into Arm 2. Participants in Arm 1 will be randomly assigned to receive usual care (control), the alcohol intervention, or the sexual health intervention. Participants in Arm 2 will be randomly assigned to receive either usual care (control) or the teen driving intervention.

3.3 Study Duration, Enrollment and Number of Sites

3.3.1 Duration of Study Participation

Arm 1 – Approximately 4-5 months from the time of enrollment.

Arm 2 - Approximately 6-7 months from the time of enrollment.

3.3.2 Total Number of Study Sites/Total Number of Subjects Projected

The study will be conducted within participating clinical sites in the CHOP PeRC network. Approximately 900 total subjects will be enrolled to produce 375 evaluable subject dyads (375 adolescents and 375 parents). Additionally, approximately 15 health care providers will be enrolled to produce 10 evaluable health care provider subjects. Finally, approximately 10 research assistants (health coaches) will be enrolled to produce 5 evaluable subjects.

3.4 Study Population

3.4.1 Inclusion Criteria

<u>Adolescents</u>

1) Males or females age 14 to 17 years at the time of their upcoming well-child visit OR at the time their medical certification form for the permit application is completed.
2) CHOP primary care patient.
3) Scheduled for a well-child visit that parent and teen both plan to attend OR had their medical certification completed in the past 8 weeks by a health care provider at a CHOP Primary Care Practice
4) *[For Arm 2 only]* Planning on having the medical certification for the permit application completed at their next well child visit OR had their medical certification completed in the past 8 weeks by a health care provider at a CHOP Primary Care Practice and planning on taking their driving permit test in the next 8 weeks.

<u>Parents</u>

1) Parent or legal guardian of a male or female age 14 to 17 years at their upcoming well-child visit at a CHOP primary care practice or at the time the teen's medical certification form for the permit application was completed.

<u>Health Care Providers</u>

1) Health care provider employed at a CHOP PBRN primary care site participating in the study (IRB 15-011732)

Research Assistant / Health Coach

1) Research staff member listed on the protocol (IRB 15-011732)

2) Trained by PI to participate in health coach activities

3.4.2 Exclusion Criteria

Adolescents

1) Not fluent in written or spoken English.

2) Attending a *new* patient well-child visit.

3) Developmental Delay or Pervasive Developmental Disorder.

4) Pregnant female.

5) Already has driving permit (has taken and passed the knowledge test).

Parents

1) Not fluent in written or spoken English.

Health Care Providers

None

Research Assistant / Health Coach

None

Subjects that do not meet all of the enrollment criteria may not be enrolled. Any violations of these criteria must be reported in accordance with IRB Policies and Procedures.

It is possible that more than one eligible teen from a given family will be eligible to enroll, as families often schedule appointments back to back. In these instances, only one parent-teen dyad per household will be enrolled. The decision on who to enroll will be selected based on the following criteria: 1) If there is an adolescent aged 16-17 that is eligible for Arm 2, we will prioritize recruitment of this dyad; 2) If we are unable to recruit this dyad, then we will randomly select from the remaining eligible adolescents.

4 STUDY PROCEDURES

4.1 Screening Visit (T=0)

The study screening phase involves a letter being given or mailed home to describe the study to all eligible participants, verbal consent to screen (waiver of assent is requested), verbal informed consent and assent over the phone. After participants are enrolled, they will be randomized into a study group. Then we will administer a brief baseline intake survey to parents and teens separately over the phone and/or we will send it electronically to collect sociodemographic information, baseline parenting and parent-teen communication and relationship variables, baseline cognition variables, and baseline assessment of health behavioral variables.

The following procedures will take place during the phone screening visit:

- Verbal Consent to Screen (Waiver of Assent Requested)
- Informed Consent and Assent
- Randomization
- Administration of Baseline Screening Intake Assessment with parent and teen
- Email electronic versions of the intervention materials to intervention group parents (Arm 2 only)

4.2 Study Intervention Phase

The study intervention phase consists of written materials for parental review, an RA orientation at the child's well-child visit or over the phone, a physician endorsement given during the child's well-child clinic appointment or mailed to the home, and a booster phone call from the RA approximately 2 weeks after the initial orientation of the intervention materials.

4.2.1 Intervention Materials

Intervention materials are shown in Appendix A-D. Arm 1 participants in the intervention groups will receive a parenting handbook (Appendix A) that focuses on how to communicate effectively and respectfully with teenagers. It contains written information and activities. Depending on which group the patient is randomized to, parents will also receive an additional module and tip sheet on one of three health behaviors: (1) sexual health (Appendix B); (2) alcohol prevention (Appendix C); and (3) teen driving (Appendix D). Materials will be sent electronically to intervention group parents (Arm 2 only) after enrollment and will be provided in hardcopy to intervention group parents at the teen's well-child appointment if applicable. Parents in the control group will be emailed or mailed intervention materials at the end of the study when all surveys are complete.

4.2.2 Clinic Visit (Intervention subjects only) (T=1)

Intervention group parents will be greeted at the clinic prior to their child's well-child appointment by a member of the research team. In a private location the parent will receive

a brief orientation to the materials by a trained RA health coach on the topic to which they've been assigned. This discussion will typically happen while the teen is having their well-child visit. At the end of the well-child examination the parent and teen will meet briefly with the HCP who will endorse the materials and provide key health and safety messages using a brief Provider Endorsement Sheet (see Appendix E). The parent will leave the appointment/visit with an agreed-upon date scheduled to talk to the teen about the health topic (driving, sexual health, alcohol) and with a "prescription" for reading/using the materials and talking with the teen provided by the HCP (see Appendix E). Arm 2 intervention group parents whose teens do not have a scheduled well-child visit and are a part of the study because they had their medical certification form completed by a health care provider at a CHOP Primary Care Practice in the last 8 weeks will receive the intervention materials and provider "prescription" via mail and will receive an orientation of the materials by a trained RA health coach over the phone.

Parent and teen participants randomized to the control groups will receive care as usual at their well-child visit. RAs will conduct a chart review to document whether or not the well-child visit was completed (date/time).

4.2.2.1 RA Orientation

- Greet family, move with parent to confidential/quiet location
- Confirm Assignment Status
- Provide orientation to intervention materials with parent, including discussion of "The Basics" materials
- Give parent hardcopy of materials
- Bring parent to the exam room with teen and HCP
- Give HCP indication of assignment status (color coded file folder)
- Complete clinic implementation fidelity checklist (Appendix F)

4.2.2.2 Health Care Provider Endorsement

- Provide key messages (Appendix E) to parent and teen
- Provide intervention prescription (Appendix E) to parent

4.2.3 Booster Call and T2 Survey (Intervention Parents only) (2 weeks) (T=2)

4.2.3.1 Booster Call

Approximately 2 weeks (-3 days/ + 2 weeks) after the well-child visit or health coaching phone call (arm 2), a trained RA health coach will place a "booster" phone call to the intervention group parents to finish reviewing materials / content that might not have been completed at the clinic visit and to discuss questions and concerns that the parent might have. Much of the flow of conversation will be guided by the individual parent. If parents have not talked to the teen, the RA will discuss barriers to this happening and will initiate another booster call in approximately 2 weeks. This process will be repeated up to 3 times (i.e., booster calls will be repeated every 2 weeks for a total of 3 possible calls).

The following procedures will take place during the Booster call:

- RA places call to parent and has discussion about materials and the status of the parent-teen talk

- If parent has NOT talked to the teen, RA will discuss strategies to overcome barriers and continue to place calls every 2 weeks, up to 3 total calls
- Complete booster call implementation fidelity checklist (Appendix F).

4.2.3.2 T2 Survey

The 2 week survey will be given to the parents in the intervention groups and can be administered at the time of the booster call or can be scheduled for another date to be completed via phone or via secure web-survey.

4.2.4 Final Follow-Up Survey (approximately 4-6 months) (T=3)

Approximately 4-5 months after the well-child visit, we will administer a parent and teen follow-up survey to all Arm 1 participants over the phone or via secure web-survey.

Approximately 6 months after the well-child visit or health coach phone call (for Arm 2 form drop off group), we will administer a parent and teen follow-up survey to all Arm 2 participants over the phone or via secure web-survey.

4.2.5 Health Care Provider Feedback Survey

After recruitment has ended, HCPs enrolled in the project will be asked to provide feedback about their “P4 Study” experience via an anonymous survey. HCPs will be made aware that the survey is part of the research study and by completing the survey they are giving their consent. It’s important that the surveys are completed anonymously so the HCPs feel comfortable sharing any negative feedback about research personnel and/or practice leadership.

HCPs will have the option to complete the survey electronically (REDCap) or via hard copy (paper and pencil). Providers who choose to complete the paper and pencil version of the survey will be asked to place their completed surveys in a lock box/bin at the practice. Surveys will then be transferred via lockbox to 3535 Market Street where they will be entered into REDCap and stored in a locked filing cabinet.

4.3 Subject Completion/Withdrawal

Subjects may withdraw from the study at any time without prejudice to their care. They may also be discontinued from the study at the discretion of the Investigator for lack of adherence to study treatment or visit schedules, or AEs. The Investigator may also withdraw subjects who violate the study plan, or to protect the subject for reasons of safety or for administrative reasons. It will be documented whether or not each subject completes the clinical study. If the Investigator becomes aware of any serious, related adverse events after the subject completes or withdraws from the study, they will be recorded in the source documents and on the CRF.

5 STUDY EVALUATIONS AND MEASUREMENTS

5.1 Screening and Monitoring Evaluations and Measurements

See Table 3 for a summary of T0-T3 measures. All assessment materials for all study arms and groups can be found in Appendices H-K.

Table 3: Measure Summary for T0-T3 Surveys

	**T0 Baseline Intake**		**T1 Clinic/Phone Intervention**	**T2 2 Week Follow Up**		**T3 Long-Term Follow Up**	
**Measures**	**T0 Parent (I + C)**	**T0 Teen (I+C)**	**T1 RA**	**T2 RA**	**T2 Parent (I)**	**T3 Parent (I+C)**	**T3 Teen (I+C)**
**Intervention Fidelity Assessment**			x	x	x		
**Intervention Acceptability**			x	x	x	x	
**Intervention Feasibility**			x	x			
**Sociodemographics**	x	x					
**Social Desirability (Crowne Marlow)**	x	x					
**Relationship Characteristics**							
Child disclosure	x	+					
Parental Solicitation	x	+					
Parental Control	x	+					
Feeling Controlled		x					
Quality of Relationship	x	x					
Perceived Trustworthiness (general)	x	x					
Parenting Style		x					
Parental Knowledge (general)		x					
Perceived Family Support							^
**Cognitive variables**							
Perceived HCP norms						^	^
Attitudes/Beliefs							
Communication						^	x
Parental Approval (ALC; Sex; TD)	x	x				^	x
Knowledge						X	x
Self-efficacy						^	X
**Communication**							
General (PACS) (Olsen)	x	x				^	^
PACS-STD/HIV (Sales 2008)							^
Strengths	x	x				x	X
Topic Specific-Quantity (ALC, Sex)						^	x[1]
Topic-Specific Content (ALC, Sex)						^	X
Communication Intentions						^	
**Behavior**							
Parent (TD, ALC, Sex)	x					+	
Parental perception of teen (TD,ALC, Sex)	x					X	
Teen (TD, ALC, Sex)		x					X[1]
**Behavioral Intentions** (ALC, Sex)							^
**HCP Relationship**							
Future Visits						^	^

*Note: ALC=alcohol use; C= control; HCP= healthcare provider; I=intervention; PACS= Parent Adolescent Communication Scale; TD= teen driving; sex=sexual behaviors; ^=Arm 1 only; +=Arm 2 only; x=both Arm 1 and Arm 2
[1]selected alcohol/sex questions will be asked of Arm 2 for exploratory purposes

5.1.1 Confirmation of eligibility

Members of the research team will securely document eligibility and consent for all subjects on hardcopies and retain the data directly in the REDCap electronic data capture system and/or locked filing cabinet.

5.1.2 Contact Information

The following contact information will be collected from both parents and teens (unless otherwise specified) (Appendix H):

- First Name
- Last Name
- Home Address
- Zip Code
- Primary Phone Number
- Secondary Phone Number
- Additional Phone Number, if available
- Email Address
- Additional Email address, if available
- Contact preferences (e.g., time of day and mode)
- Date of Birth
- Learner Permit Number, if applicable (teens only)

After collecting the information, the research team will assign each subject a unique identification (UID) number. This UID will serve to code all subsequent data collection tools.

5.1.3 Baseline Screening Intake Assessment (T0)

The below information will be collected from parents and teens at the baseline assessment (see Appendix I).

Parents will be asked to complete a secure web-survey or phone interview assessing:

- Parent Socio-demographics: Relationship to patient; Gender; Ethnicity; Race; Current marital status; Highest degree of education obtained.
- Relationship Characteristics (Stattin & Kerr, 2000; Kerr & Stattin, 2000): Child disclosure; Parental solicitation; Parental control; Quality of relationship with teen; Perceived general trustworthiness.
- Cognitive variables: Parental approval of adolescent behaviors.
- Communication Variables (Olsen; Crosby) General parent-teen communication; Communication about strengths.
- Parent behaviors: Driving; Alcohol.
- Parent perceptions of adolescent behaviors: Alcohol; Sexual behaviors; Driving.
- Social Desirability (Crowne-Marlow).

Teens will be asked to complete a secure web-survey or phone interview assessing:

- Adolescent Socio-demographics: Sex; Ethnicity; Race; Grade in school.
- Relationship Characteristics (Stattin & Kerr, 2000; Kerr & Stattin, 2000): Feeling controlled; Quality of relationship with parent; Perceived general trustworthiness; Parenting style; Parental general knowledge. Child disclosure; Parental solicitation; Parental Control (Arm 2).
- Cognitive variables: Perceived parental approval of adolescent sexual/alcohol adolescent behaviors.
- Communication Variables (Olsen) General parent-teen communication; Communication about strengths.

- Adolescent behaviors: Driving; Alcohol; Sexual behaviors.
- Social Desirability (Crowne-Marlow).

5.1.4 Health Coach Acceptability, Feasibility, and Fidelity Assessment

A fidelity checklist will be completed by the RA Health Coach at the clinic visit (T1) and booster call (T2) (see Appendix F). Items covered in the Health Coach Fidelity Assessment are listed below. All data is collected by RA report, unless otherwise indicated.

- Health Coach sociodemographics: age, gender, ethnicity, race, degree of education (T1 and T2) (collected via HC survey)
- Health Care Provider sociodemographics: age, gender, ethnicity, race, role/type, years in practice (collected via HCP survey)
- Health Care Provider familiarity with patient (# of visits between HCP and patient in past 5 years), # of patients seen per hour day of visit (T1) (collected via chart review by RA)
- Participant ID (T1 and T2)
- Visit date/time/length (T1)
- Checklist of intervention materials distributed in clinic (T1)
- Checklist of intervention items distributed by health care provider during clinic visit (T1)
- Checklist of intervention items covered by health coach in clinic (T1)
- Checklist of intervention items covered by health coach in booster call (T2)

Of note, further information to assess intervention fidelity will be collected from parents in the T2 Survey to confirm RA reports of intervention fidelity.

In addition, all RAs will keep a running journal while at the clinic recording comments by clinic staff about intervention implementation issues, and all observations related to acceptability and feasibility of intervention implementation (T1). RAs will also record comments made by parents related to acceptability and feasibility of intervention during the 2-week booster call / survey (T2).

5.2 Efficacy Evaluations

5.2.1 2 Week Survey [2 weeks post clinic visit (- 3 days/ + 2 weeks)] (T2)

Parents in the intervention groups will be administered a 2 week follow up survey containing the below items (Appendix J):

- Intervention Fidelity: Query about which materials parents received at the clinic
- Topic-specific Communication: how much did parent talk with the teen about the target behavior; when/how did talk take place and how did it go; Barriers to talking and methods for overcoming barriers; reasons for not having the talk and plans for having the talk in the future
- Intervention Acceptability: did parent use materials; helpfulness of materials
- Parent perceptions of adolescent participation and disengagement in their recent well-child visit

5.2.2 Final Follow-Up Survey [4-6 months post clinic visit/health coach phone call] (T3)

The below items will be assessed at the final follow-up with all parents and teens (see Appendix K).

Parents will be asked to complete a secure web-survey or phone interview assessing:

- Cognitive Variables: Attitudes about parent-teen communication (Arm 1); Parental approval of adolescent sexual/alcohol behaviors (Arm 1); Parent knowledge of sexual health/alcohol related information (Arm 1) or teen driving safety information (Arm 2); Communication self-efficacy (Arm 1); Perceive HCP norm regarding parent-teen communication about sexual/alcohol behaviors (Arm 1).
- Communication Variables: General parent-teen communication (Arm 1); Communication about strengths; Parent-teen communication quantity and content about sexual health/alcohol (Arm 1) or teen driving (Arm 2); Communication intentions (Arm 1).
- Behavior: Parental perceptions of teen behavior; Parent practice driving behaviors (Arm 2)
- Communication Intentions. Intentions to communicate about sex/alcohol (Arm1).
- HCP Relationship: Likelihood of future visit to HCP for routine, sexual health, alcohol-related issues (Arm 1).
- Intervention Acceptability.

Teens will be asked to complete a secure web-survey or phone interview about:

- Relationship Characteristics: Perceived family support (Arm 1).
- Cognitive Variables: Perceived parental approval of adolescent sexual/alcohol adolescent behaviors (Arm 1) and driving safety (Arm 2); Teen knowledge of sexual health/alcohol related information (Arm 1) or teen driving safety information (Arm 2); Self-efficacy related to sexual/alcohol behaviors (Arm 1); Perceived HCP norm - communication about sexual/alcohol behaviors (Arm 1).
- Communication Variables: General parent-teen communication (Arm 1); Communication about strengths; Parent-teen communication quantity and content about sexual health/alcohol (Arm 1 and selected sexual/alcohol questions for Arm 2) or teen driving (Arm 2).
- Behavior: Teen behavior sexual/alcohol (Arm 1 and selected sexual/alcohol questions for Arm 2) or driving (Arm 2).
- Behavioral Intentions. For teens not engaging in target sexual/alcohol behaviors, intentions will be assessed (Arm1).
  HCP Relationship: Likelihood of future visit to HCP for routine, sexual health, alcohol-related issues (Arm 1).

5.2.3 Healthcare Provider Feedback Survey

The below items will be assessed at the Health Care Provider Feedback Survey with all HCPs who opt to complete the survey (see Appendix N).

- How things went during the study
- Suggestions for changes/future studies

- Impact on value of pediatric primary health care
- How to implement interventions like these in real life practice
- Any other helpful feedback

5.3 Safety Evaluation

This is a minimal risk, behavioral intervention study. There are no expected serious adverse events related to the intervention or participation in this study. Any psycho-social risks are minimal and unlikely and will be monitored by reviewing and evaluating parent and teen periodic reports and by the timely reporting of any adverse events. As with any research project, there is a risk to loss of confidentiality. There are safeguards in place to protect confidential information including maintaining the master list in REDCap due to the internal controls already in place, keeping PHI separate from study data, and entering data directly into a secure database. The study team has received a Certificate of Confidentiality from the National Institutes of Health to further protect subject confidentiality.

As this study involves questionnaires related to sexual behavior, it is possible that research subjects will spontaneously disclose information related to statutory sexual assault. In this unlikely instance, the RA will immediately notify the PI. If it is a teen that made the disclosure, the PI will make an assessment of the situation in collaboration with CHOP's social work department in order to determine appropriate next steps and reporting requirements. If it is a parent that made the disclosure, the RA will immediately notify the PI who will make an assessment and direct the parent to their child's primary care physician for follow-up. The PI will educate the RAs on Pennsylvania's mandatory reporting requirements prior to conducting the trial.

6 STATISTICAL CONSIDERATIONS

This is a pilot efficacy and implementation study. As such, some analyses may be underpowered and all analyses can be considered exploratory.

6.1 Primary Endpoint

The primary endpoints are evaluating the difference between the intervention and control groups on adolescent health behaviors (alcohol, sexual, driving behaviors) and behavioral intentions (alcohol/sexual behavioral intentions).

6.2 Secondary Endpoints

Secondary endpoints:

- Reach: The proportion of eligible participants who agreed to participate in the intervention; of those that participated: the proportion of participants who read the materials, and talked with their teen about the indicated health topic.

- Feasibility of delivery in high volume clinic practice: Determine implementation quality (% of intervention components delivered with fidelity) and determine their association with clinic-level (e.g., patient volume) and provider-level factors (e.g., years of experience). These endpoints will be assessed via process measures.

- Evaluate the difference between the treatment and control conditions on:

  - Parent-Teen Communication: General parent-teen communication; Topic-specific quantity and content of parent-teen communication (sexual/alcohol/ driving/strengths); Parents' communication self-efficacy; Parents' communication intentions)
  - Parent and Teen Topic-Specific Knowledge: Teen driving/sexual health/alcohol)
  - Adolescent Perceptions of Parental Attitudes: Perceived parental disapproval of the teen having sex, drinking, and engaging in risky driving; Perceptions of the parent as a trustworthy source of information on sex, drinking/alcohol use, and teen driving
  - HCP Relationship: Parent and teen perception of the importance HCP places on parent-teen communication; Parent and teen perception of HCP as resource for future routine, sexual health, and alcohol-related issues

6.3 Statistical Methods

6.3.1 Baseline Data

Baseline and demographic characteristics will be summarized by standard descriptive summaries (e.g. means and standard deviations for continuous variables such as age and percentages for categorical variables such as gender).

6.3.2 Efficacy Analysis

The primary analysis will be based on an intention to treat approach. We will use descriptive and inferential statistical techniques. We will conduct preliminary analyses to make sure that the data are reasonable and to identify outliers.

Descriptive statistics, such as means, standard deviations, medians and range for continuous variables and percentages for categorical variables will be generated for all quantitative data of the overall sample and by condition (treatment vs. control). Relevant continuous measures will be evaluated for normality and those differing markedly from normality will be summarized by using medians, interquartile ranges, and box-plots. Histograms will be the graphical measure used to complement descriptive analyses of categorical data.

For statistical inference, we will employ a variety of parametric and non-parametric statistical techniques such as t-test, chi-square, Fisher's exact test, linear and non-linear regression analysis, random effects models, generalized estimating equations structural equation modeling, will be used as needed. Some of the regressions may have to be run in the stepwise mode, with regression used in some steps to perform covariate adjustments of between-group comparisons. In other analyses, the regressions would be used as part of propensity analysis. Missing data approaches will be used. Statistical tests will be two tailed with an alpha = 0.05.

Note that as this is a pilot study, we realize that we may be underpowered for some analyses. The conduct of these analyses remain important so that we can identify practice-level effect sizes for the variables of interest.

6.3.3 Safety Analysis

All subjects randomized to receive an intervention (sexual health; alcohol; driving) will be included in the safety analysis. This is a minimal risk study and as such AEs and SAEs are not expected. However, AE incidence will be monitored and reported as necessary.

6.4 Sample Size and Power

This is a pilot efficacy and implementation study. With this in mind we conducted an initial power analysis using observed effect sizes from prior RCTs of intervention materials (in non-clinic settings). Effect size estimates were taken from the TeenDrivingPlan RCT; treatment (n=86) vs control (n=65) (See Mirman et al., 2014 JAMA Peds) and from the Families Talking Together RCT; treatment (n=124) vs control (n=126) (see Guilamo-Ramos et al., 2010).

Teen Driving Behavioral Outcomes

- The average amount of practice quantity (0 hrs. = 1; <1 hr = 2; 1 to 2hrs = 3; 3 to 5 hours = 4; 6 to 10 hours = 5; > 10hrs = 6) across environments/conditions (parking lot, residential, highway, intermediate roads, rural roads, night, and bad weather). Effect sizes ranged from: (d=.50 to d=.34)
- The number of environments and conditions where the teen practiced for at least 1 to 2 hours: TX: Med(IQR) = 6 (5,6) vs. Control: 5 (4,6).
- Teen-perceived support (5 pt scale), mean (SD): TX: 4.11 (.73) vs. Control: 3.78 (.79); (d=.43)
- Parent engagement (5 pt scale), mean (SD): TX: 3.51 (.54) vs. Control: 3.32 (.55); (d=.34)

*The initial TDP trial found a 9% absolute risk reduction in the termination of on-road driving assessments, but we will not be administering on road driving assessment in the pilot study.

Sexual Health Behavioral Outcomes

- Vaginal sex: TX 7% to 7% vs Control 6% to 22% (risk difference -15%)
- Freq of vaginal sex in past 30 days ((1 = never, 2 = once, 3 two or three times, and 4 = once a week or more): TX 1.00 to 1.08 vs Control 1.00 to 1.53; (d=.5)
- Engaged in oral sex (proportion y/n): TX 3% to 4% vs Control 3% to 10% (risk difference -6%)

Assuming a medium effect size (d=.5), desire to evaluate a difference between two independent means (two groups), an alpha of .05, power = .80, we will need 64 evaluable participants in each group. Assuming a smaller effect size of d=.34 the sample size increases to 137 in each group. To evaluate the difference in proportions (e.g., a 15% reduction in vaginal sex) using fisher's exact test, an alpha of .05, power = .80 we need 94 evaluable participants in each group.

Given the pilot nature of this project these are acceptable in terms of establishing initial feasibility and proof of concept.

7 PATIENTS, PARENTS AND PROVIDERS PARTNERING TO PROMOTE ADOLESCENT HEALTH (P^4) INTERVENTION

7.1 Description

This is a clinic based psychoeducational intervention for adolescent patients and their parents to improve parent-teen communication about important health topics. The intervention is designed, if possible, to coincide with the adolescent patients' well-child visits or providers' completion of adolescent patients' driver's license medical certification form and consists of the following components: (1) In-person or over the phone orientation session with a trained RA Health Coach (HC) and parent, (2) Distribution of psychoeducational materials to the parent, (3) Endorsement and delivery of key messages from the health care provider, and (4) "Booster" phone call placed by the RA HC.

7.1.1 Trained RA Health Coaches (HC)

HCs will be members of the study team trained by the PI and Project Director. They will meet with parents of teens at the time of the teen's well-child visit or over the phone to provide an orientation of the materials. HCs will provide hardcopies of the psychoeducational materials (section 7.1.2), deliver key messages, and work with the parent to identify a date and time to talk with the teen about the targeted health behavior using the "Basics" (see section 7.1.2 below) as a guide. The HC will place a booster phone call to the parent approximately 2 weeks after the clinic visit to re-deliver key messages, cover additional content as applicable, and discuss how the conversation with the teen went, providing support as needed.

The goal of the HC conversations (face-to-face and phone calls) is to provide parents with the knowledge and skills to have meaningful and accurate information on important adolescent health topics and to motivate parents to speak with their teen about these topics by increasing self-efficacy and decreasing perceived barriers.

7.1.2 Intervention Materials

The materials consist of a Parent-Teen Communication Handbook that includes 7 modules on cultivating a positive parent-teen relationship and information on adolescent health and development. It also includes a workbook for structured communication activities *"Things to do and things to talk about".* This is intended to serve as a "desk reference" for parents and is not necessarily something that they would be expected to read cover-to-cover.

This information is complemented by stand-alone content specific modules on sexual health, alcohol prevention, and teen driving. Each behavior specific module has a brochure-style handout called "The Basics". The Basics provide parents with the essential information that they need to have a productive discussion with their teen about these important health behaviors. For the teen driving module we will use the *TeenDrivingPlan Parent Guide* in lieu of creating a new "Basics" brochure for teen driving. See Appendices A-D.

7.1.3 Endorsement by Health Care Provider

If intervention coincides with well-child visit, the health care provider will deliver key messages to the parents and teens and endorse the materials verbally, as well as document it on the health care provider endorsement sheet, and in the form of a written "prescription" (Appendix E). The written prescription can be mailed if intervention takes place over the phone.

SAFETY MANAGEMENT

7.2 Clinical Adverse Events

Clinical adverse events (AEs) will be monitored throughout the study by the PI.

7.3 Adverse Event Reporting

Since the study procedures are not greater than minimal risk, SAEs are not expected. If any unanticipated problems related to the research involving risks to subjects or others happen during the course of this study (including SAEs) they will be reported to the IRB in accordance with CHOP IRB SOP 408: Unanticipated Problems Involving Risks to Subjects. AEs that are not serious but that are notable and could involve risks to subjects will be summarized in narrative or other format and submitted to the IRB at the time of continuing review.

8 STUDY ADMINISTRATION

8.1 Treatment Assignment Methods

8.1.1 Randomization

A table of random numbers will be used to allocate subjects into each arm of the study. At screening/enrollment research staff will access an electronic database with the allocation information for each Arm.

8.2 Data Collection and Management

We will use REDCap for direct and secondary data entry and electronic survey data distribution and collection. REDCap will also be used for tracking of study procedures (e.g. consent/assent and eligibility) after having been documented on paper forms. Secondary data entry in REDCap will take place if HCPs chose to complete a paper and pencil version of the Health Care Provider Feedback Survey. HCPs will be asked to place their completed anonymous surveys in a lock box/bin at the practice. Only members of the study team will be able to access the completed Provider Feedback Surveys in the lock box. Surveys will then be transferred via lockbox to 3535 Market Street where they will be entered into REDCap and stored in a locked filing cabinet. REDCap has security features in place to protect the confidentiality of subject data. Datasets will be stored on a secure shared drive that only members listed on the protocol can access. All data exports for analysis will be completely de-identified as the study data will not be collected with any identifying information. All identifying information will be stored in a separate REDCap tracking database and will not be exported with the study data being used for analysis. Any paper forms will be stored in a locked file cabinet in the PI's administrative offices.

We will protect subject confidentiality and ensure the data are secure in the following ways:

1) Each subject will be assigned a unique identifier (UID) used in all data collection.
2) Subject contact information will be stored in a secure REDCap database. Access to the identifiable data in REDCap will be limited to study team members.
3) All files, forms, databases, and printouts will be available only to staff working on the study. All research staff will be CITI certified.

8.3 Confidentiality

No identifiable data will be used for future study without first obtaining IRB approval. The investigator will obtain a data use agreement between the provider (the PI) of the data and any recipient researchers (including others at CHOP) before sharing a limited dataset.

8.4 Regulatory and Ethical Considerations

8.4.1 Data and Safety Monitoring Plan

This is a minimal risk study. Dr. Carol Ford, as the Principal Investigator, will be responsible for monitoring the safety of study subjects and complying with all reporting requirements. She will report any adverse events immediately to the CHOP IRB and to the sponsoring agency, if indicated by the IRB.

8.4.2 Risk Assessment

This is a minimal risk study consisting of the completion of self-report surveys, and receiving individualized coaching on topics that parents have previously identified as important and valued (Ford et al., unpublished data). Further, prior evaluations of these materials have demonstrated effectiveness in other settings. The control condition is usual practice / standard care with which there are no known risks (i.e., adolescents in the control group will receive the usual care typically provided to adolescent patients at the well-child visit). There are no physical or economic risks. The learning-to-drive period is the safest period for new drivers and no safety negative outcomes have ever been observed in our prior research with this population of learner drivers.

8.4.3 Potential Benefits of Trial Participation

We will not guarantee direct benefits associated with this study. However, we are utilizing evidenced-based interventions that have been shown to improve parent-teen communication and impact teen health behaviors. Therefore, participants may benefit from increases in parent communication skills, improved parent-teen-provider relationship dynamics, and positive health outcomes for the adolescent. Indirect benefits include participating to the development of effective adolescent healthcare materials.

8.4.4 Risk-Benefit Assessment

Because the study is minimal risk and there are potentially both direct and indirect benefits associated with participation, the benefits outweigh the risks.

8.5 Recruitment Strategy

Prospective participants will be identified via EPIC. Parents of adolescent patients that will be 14-17 years of age at their upcoming scheduled well-child visit at a participating CHOP Primary care site will receive a letter in the mail describing the study. Additionally, 16-17 year old patients will be identified by the participating primary care practice when patients drop off their medical certification form for the permit application to be signed by their CHOP primary care provider. A letter inviting the family to participate in the project will be given to the family when their completed medical certification form is returned. The letter will include study contact information and if a family is interested in getting more information or in participating they can call or email to inquire. All participants that do not reach out after receiving the study letter will be called by a member of the study team to describe the study and gauge interest in participating.

8.6 Verbal Consent to Screen & Informed Consent/Assent and HIPAA Authorization

All interested parental participants will be asked to complete a brief verbal consent to screen for eligibility over the phone. We have requested a waiver of assent for the verbal consent to screen, as we will be able to obtain all relevant information from the parent directly. However, we will still collect verbal assent from the teens for the main study procedures.

If participants are interested and meet all eligibility criteria, a member of the research team will collect parental consent and teen assent, as well as HIPAA Authorization over the phone prior to their well-child visit appointment. The study team member will speak to parents first, followed by teens. After speaking with parents we will ask that the teen to move to a quiet and private location in the home. Potential participants will be given as much time as they need to ask questions.

It is possible that subjects who are 17 years of age at the time of enrollment could turn 18 during the 6-7 months of study participation. Because we are collecting DOB, subjects that will turn 18 over the course of the study will be identified during the baseline data collection visit. At this time, the RA will schedule a follow-up phone call to re-consent these subjects when they turn 18. During the re-consent call, the RA will explain that because the subject has turned 18 years old he/she is able to consent to participation in the research study for him/herself. The RA will proceed with the informed consent process as outlined above.

8.6.1 Waiver of Documentation of Consent and Partial Waiver of HIPAA

This research study meets the criteria for wavier of documentation of consent/assent under #46.117(c) (2). The research presents minimal risk to participants and involves no procedures for which written consent is normally required outside of the research context.

All subjects will be given a study information letter (Appendix L); letters will be given in person at their well-child visit, mailed home, or emailed based on the families' preference after enrollment. As we are randomizing prior to the clinic visit it is too late to screen and consent at the time of the visit. We have kept research procedures (consent, surveys) separate from in office procedures so as not to disrupt the flow of the clinic and to gauge whether or not the intervention can be implemented with fidelity during normal clinical operations (see Secondary Aim section 6.2).

Additionally, a waiver of documentation of consent is requested for the completion of the Health Care Provider Feedback Survey. The survey introduction contains language denoting that by proceeding with the survey the health care provider is giving their consent to participate.

8.7 Payment to Subjects/Families

All parents will be compensated $10 for completing the Intake Survey and $20 for completing the final follow-up survey. Intervention group parents will be compensated $5 for completing the 2 week survey.

All adolescents will be compensated $10 for completing the Intake Survey and $20 for completing the final follow-up survey.

If subjects do not complete all study procedures, they will be compensated less in proportion to the number of activities completed.

9 PUBLICATION

CHOP investigators will have complete access to all study data and will publish results of the study in the form of manuscripts which will be submitted to the peer-reviewed scientific literature in journals relevant to the topic area.

10 REFERENCES

Centers for Disease Control (CDC). Youth Risk Behavior Surveillance — United States, 2013. *Morbidity and Mortality Weekly Report Surveillance Summaries.* 2013;63(4):1-168. Accessed February 18. 2015: http://www.cdc.gov/mmwr/pdf/ss/ss6304.pdf

Centers for Disease Control (CDC). Diagnoses of HIV infection and AIDS in the United States and dependent areas, 2009. *HIV Surveillance Report.* 2009;21:1-79. Accessed February 18, 2015: http://www.cdc.gov/hiv/pdf/statistics_2009_HIV_Surveillance_Report_vol_21.pdf

Sales JM, Milhausen RR, Wingood GM, DiClemente RJ. Validation of a Parent-Adolescent Communication Scale for Use in STD/HIV Prevention Interventions. Health Education & Behavior, Vol 35(3); 332-345 (June 2008.

Ford CA, Cheek C, Culhane J, Fishman J, Mathew L, Salek EC, Webb D, Jaccard J. Parent and Adolescent Interest in Receiving Adolescent Health Communication Information From Primary Care Clinicians. Manuscript in preparation.

Ford CA, Davenport AF, Meier A, McRee AL. Partnerships between parents and health care professionals to improve adolescent health. *J Adolesc Health.* 2011;49(1):53-57.

Guilamo-Ramos V, Jaccard J, Turrisi R, Johansson M. Parental and School Correlates of Binge Drinking Among Middle School Students. *American Journal of Public Health* 2005;95(5).

Guillamo-Ramos V, Bouris, Jaccard J, et al. A Parent-based Intervention to Reduce Sexual Risk Behavior in Early Adolescence: Building Alliances Between Physicians, Social Workers, and Parents. *J Adolesc Health*. 2011 February ; 48(2): 159–163.

Hamilton BE, Martin JA, Ventura SJ. Births: Preliminary data for 2009. NATIONAL VITAL STATISTICS REPORTS 2010;59(3).

Jaccard J, Dodge T, Dittus P. Parent-Adolescent Communication about sex and birth control: A conceptual framework. *New directions for child and adolescent development* 2002;97:9-41.

Kerr M, Stattin H. What parents know, how they know it, and several forms of adolescent adjustment: Further support for a reinterpretation of monitoring. *Developmental Psychology* 2000;36(3):366-380.

Mayhew DR., Simpson DM, Pak A. "Changes in collision rates among novice drivers during the first months of driving." *Accident Analysis & Prevention.* 2003;35(5):683-691.

McCartt AT, Shabanova VI, Leaf WA. Driving experience, crashes and traffic citations of teenage beginning drivers. *Accident Analysis & Prevention.* 2003;*35*(3):311-320.

Mirman JH, Curry AE, Winston FK, et al. Effect of the Teen Driving Plan on the Driving Performance of Teenagers Before Licensure: A Randomized Clinical Trial. *JAMA Pediatrics.* Published online June 23, 2014.

Mirman JH, Albert, Curry AE, et al. TeenDrivingPlan Effectiveness: The Effect of Quantity and Diversity of Supervised Practice on Teens' Driving Performance. *Journal of Adolescent Health.* In press.

Olson, D. H.. Family inventories: Inventories used in a national survey of families across the life cycle. St Paul, MN: Family Social Science, University of Minnesota. 1985

Stattin H, Kerr M. Parental monitoring: A reinterpretation. *Child Development* 2000;71(4):1072-1085.

Turrisi, Jaccard, Taki, et al. Examination of the Short-Term Efficacy of a Parent Intervention to Reduce College Student Drinking Tendencies. *Psychology of Addictive Behaviors* 2001;15(4):366-372.

Weinstock H, Berman S, Cates W. Sexually transmitted diseases among American youth: incidence and prevalence estimates, 2000. PERSPECTIVES ON SEXUAL AND REPRODUCTIVE HEALTH 2004;36(1):6-10.

Winston FK, Mirman JH, Curry AE, et al. Engagement with the TeenDrivingPlan and diversity of teens' supervised practice driving: lessons for internet-based learner driver interventions. Inj Prev. Published Online First June 20, 2014. doi:10.1136/injuryprev-2014-041212

Zimet G, Dahlem NV, Zimet SG, Farley GK. The multidimensional scale of perceived social support. Journal of Personality Assessment. 52;30-41. 1988.

APPENDIX

A: Parenting Educational Materials

B: Sexual Health Educational Materials

C: Alcohol Prevention Educational Materials

D: Teen Driving Educational Materials

E: Provider Endorsement & Prescription

F: Health Coach Fidelity Assessment

H: Contact Surveys

I: Intake Baseline Surveys

J: 2 Week Follow-Up Survey

K: Final Follow-Up Survey

L: Study Information Letter

M: Resource List

N: Health Care Provider Feedback Survey
