# Supplementary material for: Effect of Primary Care Parent-Targeted Interventions on Parent-Adolescent Communication About Sexual Behavior and Alcohol Use: A Randomized Clinical Trial
Source: JAMA Netw Open. 2019 Aug 16;2(8):e199535. doi: 10.1001/jamanetworkopen.2019.9535 (PMC6704744; doi:10.1001/jamanetworkopen.2019.9535)
Supplement: Supplement 2. — Data Sharing Statement [file jamanetwopen-2-e199535-s002.pdf]

Data Sharing Statement

Ford. Effect of Primary Care Parent-Targeted Interventions on Parent-Adolescent Communication About Sexual Behavior and Alcohol Use. *JAMA Netw Open*. Published August 16, 2019.
10.1001/jamanetworkopen.2019.9535

Data
Data available: No
